# Impaired Cuticle Functionality and Robust Resistance to *Botrytis cinerea* in *Arabidopsis thaliana* Plants With Altered Homogalacturonan Integrity Are Dependent on the Class III Peroxidase AtPRX71

**DOI:** 10.3389/fpls.2021.696955

**Published:** 2021-08-16

**Authors:** Riccardo Lorrai, Fedra Francocci, Kay Gully, Helle J. Martens, Giulia De Lorenzo, Christiane Nawrath, Simone Ferrari

**Affiliations:** ^1^Dipartimento di Biologia e Biotecnologie “Charles Darwin”, Sapienza Università di Roma, Rome, Italy; ^2^Department of Plant Molecular Biology, University of Lausanne, Lausanne, Switzerland; ^3^Section for Forest, Nature and Biomass, Department of Geosciences and Natural Resource Management, University of Copenhagen, Frederiksberg, Denmark

**Keywords:** cuticle, cell wall, plant immunity, *Botrytis cinerea*, peroxidase, plant-microbe interactions, pectin

## Abstract

Pectin is a major cell wall component that plays important roles in plant development and response to environmental stresses. *Arabidopsis thaliana* plants expressing a fungal polygalacturonase (PG plants) that degrades homogalacturonan (HG), a major pectin component, as well as loss-of-function mutants for *QUASIMODO2* (*QUA2*), encoding a putative pectin methyltransferase important for HG biosynthesis, show accumulation of reactive oxygen species (ROS), reduced growth and almost complete resistance to the fungal pathogen *Botrytis cinerea*. Both PG and *qua2* plants show increased expression of the class III peroxidase AtPRX71 that contributes to their elevated ROS levels and reduced growth. In this work, we show that leaves of PG and *qua2* plants display greatly increased cuticle permeability. Both increased cuticle permeability and resistance to *B. cinerea* in *qua2* are suppressed by loss of *AtPRX71*. Increased cuticle permeability in *qua2*, rather than on defects in cuticle ultrastructure or cutin composition, appears to be dependent on reduced epidermal cell adhesion, which is exacerbated by *AtPRX71*, and is suppressed by the *esmeralda1* mutation, which also reverts the adhesion defect and the resistant phenotype. Increased cuticle permeability, accumulation of ROS, and resistance to *B. cinerea* are also observed in mutants lacking a functional FERONIA, a receptor-like kinase thought to monitor pectin integrity. In contrast, mutants with defects in other structural components of primary cell wall do not have a defective cuticle and are normally susceptible to the fungus. Our results suggest that disrupted cuticle integrity, mediated by peroxidase-dependent ROS accumulation, plays a major role in the robust resistance to *B. cinerea* of plants with altered HG integrity.

## Introduction

The cell wall (CW) is crucial for various important aspects of plant biology, providing mechanical support to the protoplast, modulating cell growth and shape, and mediating cell adhesion and cell-to-cell communication (McCann and Roberts, 1991; Carpita and Gibeaut, 1993; Somerville et al., 2004; Humphrey et al., 2007). The composition and relative abundance of the major CW structural components, namely, polysaccharides [cellulose (the major load-bearing component), hemicelluloses, and pectins], glycoproteins, and phenolic compounds (including lignin) widely change during cell growth and differentiation (Anderson and Kieber, 2020). Moreover, the CW is a barrier against the attack of pathogenic microorganisms and herbivores (Albersheim et al., 2010). In the epidermal cells of the aerial parts of the plant, the outermost surface of the CW is in continuation with the cuticle, a multi-layered hydrophobic structure that limits the diffusion of water, regulates the exchange of gases with the environment, and provides protection against pathogens (Yeats and Rose, 2013). The major components of the cuticle are cutin, a polyester rich in C16-C18 oxygenated and interesterified fatty acids (FA) derivatives, such as hydroxy and hydroxy-epoxy substituted FAs, and glycerol (Holloway, 1982; Pollard et al., 2008). The cuticle also contains waxes, mixtures of hydrophobic material containing very long-chain fatty acids, and other secondary metabolites (Kunst and Samuels, 2009; Yeats and Rose, 2013). As for the CW components, the wax and cutin compositions of the plant cuticle widely vary among plant species, organs, and during development (Yeats and Rose, 2013).

Since the cuticle-CW continuum represents the first site of contact with the plant, microbial pathogens have evolved an array of enzymes that degrade the cuticle and CW structural components to assist penetration and colonization, obtain carbon sources, and promote leakage of nutrients from the protoplast (Ziv et al., 2018; Lorrai and Ferrari, 2021). In particular, at early stages of infection, several phytopathogenic fungi synthesize cuticle-degrading enzymes (cutinases, esterases, and lipases) that are thought to prepare the infection site both for adhesion and penetration (Deising et al., 1992; Berto et al., 1999; Nielsen et al., 2000; Garrido et al., 2012). Later, tissue penetration and invasion are assisted by microbial CW-degrading enzymes, most notably pectinases, which are among the first to be secreted by many phytopathogens (De Lorenzo et al., 1997; Lorrai and Ferrari, 2021) and are important pathogenicity factors, in particular for those microbes causing soft rot symptoms (Reignault et al., 2008). For instance, the necrotrophic fungus *Botrytis cinerea*, the causal agent of gray mold in several plant species, secretes large amounts of pectinolytic enzymes, most notably polygalacturonases (PGs), that degrade homogalacturonan (HG), a major pectic component, during the early phases of infection and are crucial for plant invasion, host adaptability, and to determine the type of symptoms caused by this pathogen (Have et al., 1998; Kars et al., 2005).

The vulnerability that follows CW injury caused by pathogen infection and other stresses can be compensated in the plant by the activation of an array of defense responses, including the strengthening of the altered CW itself (Vaahtera et al., 2019; Gigli-Bisceglia et al., 2020; Lorrai and Ferrari, 2021). Activation of defense responses occurs upon perception of exogenous microbe-associated molecular patterns (MAMPs) and/or CW-derived molecules and fragments that are released in the apoplast by microbial enzymes during infection and are recognized as endogenous damage-associated molecular patterns (DAMPs), thereby activating the so-called pattern-triggered immunity (PTI; Gust et al., 2017; Bacete et al., 2018; De Lorenzo et al., 2018). DAMPs include fragments of HG [oligogalacturonides (OGs)], released by PGs, and fragments released from cellulose and hemicellulose (de Azevedo Souza et al., 2017; Locci et al., 2019; Rebaque et al., 2021). Pre-treatment with exogenous OGs can protect *Arabidopsis* against subsequent infection with *B. cinerea* (Ferrari et al., 2007). *In vivo*, the accumulation of OGs during infection can be favored by the presence of PG-inhibiting proteins (PGIPs) that inhibit the activity of specific isoforms of pathogen PGs (Ferrari et al., 2013; De Lorenzo et al., 2018). The role of PGIPs in plant defense against *B. cinerea* and other pathogens has been extensively demonstrated (Kalunke et al., 2015). Moreover, the expression of a PG-PGIP fusion protein leads to the apoplastic accumulation of elicitor-active OGs and increased resistance to *B. cinerea* and bacterial pathogens (Benedetti et al., 2015). Cutin monomers and wax components released upon degradation of the cuticle during pathogen penetration also act as DAMPs and elicit defense responses (Serrano et al., 2014).

Alterations of CW integrity (CWI) triggered, for example, by genetically or chemically induced changes in the CW cellulose content can also be perceived by the plant through a dedicated surveillance system that leads to a set of responses that include accumulation of reactive oxygen species (ROS), ectopic lignin deposition, production of jasmonate (JA), and activation of defense responses (Ellis et al., 2002; Caño-Delgado et al., 2003; Manfield et al., 2004; Hamann et al., 2009). Changes in CWI can be perceived by *Catharanthus roseus* Receptor-Like Kinase 1-like proteins characterized by the presence of extracellular malectin-like domains (Franck et al., 2018). Among these proteins, FERONIA (FER) is capable of binding pectin *in vitro* (Feng et al., 2018) and has been proposed to act as a pectin integrity sensor, monitoring the HG status, and triggering compensatory responses (Lin et al., 2018). The alteration of CWI *per se* affects the ability of pathogens to successfully penetrate and/or colonize their host (Miedes et al., 2014; Nafisi et al., 2015a; Houston et al., 2016; Bacete et al., 2018). *Arabidopsis* mutants with defects in the cellulose synthase subunits CESA4/IRREGULAR XYLEM5 (IRX5) and CESA8/IRX1, necessary for secondary CW formation (Taylor et al., 2000, 2003), and CESA3/ISOXABEN RESISTANT1 (IXR1)/CONSTITUTIVE EXPRESSION OF VSP1, required for cellulose deposition in primary CW (Desprez et al., 2007; Persson et al., 2007), show a strong resistance to *B. cinerea* (Ellis and Turner, 2001; Ellis et al., 2002; Hernández-Blanco et al., 2007). Alterations in cuticle integrity also strongly enhance *Arabidopsis* resistance to *B. cinerea*, as observed in plants overexpressing a fungal cutinase (CUTE plants) or mutated in genes involved in cuticle biogenesis, including *LONG-CHAIN ACYL-COA SYNTHETASE2* (*LACS2*), *LACERATA* (*LCR*), and *BODYGUARD* (*BDG*; Bessire et al., 2007; Chassot et al., 2007; Tang et al., 2007).

The hormones jasmonate and ethylene play a major positive role for the resistance against *Botrytis*, whereas abscisic acid (ABA) is generally considered to act negatively (Mengiste, 2012; AbuQamar et al., 2017), since exogenous ABA enhances *Botrytis* pathogenicity, whereas mutations in genes involved in ABA biosynthesis or signaling reduce susceptibility to this fungus (Audenaert et al., 2002; Anderson et al., 2004; Asselbergh et al., 2007; L’Haridon et al., 2011). Because ABA biosynthesis and signaling are required for a normal cuticle structure and functionality (Curvers et al., 2010; Cui et al., 2016), it is possible that the role of this hormone in resistance to *B. cinere*a is mediated by its effects on the cuticle. Interestingly, *Botrytis* resistance associated with increased cuticle permeability can be genetically uncoupled from ABA sensitivity but requires a negative regulation of an ABA-dependent wound-inducible runaway cell death pathway (Cui et al., 2016).

We have previously reported that *Arabidopsis* and tobacco transgenic plants expressing an *Aspergillus niger* PG (henceforth, PG plants) have reduced de-esterified HG levels in the CW and reduced growth (Capodicasa et al., 2004; Lionetti et al., 2010; Francocci et al., 2013) and display a robust resistance to *B. cinerea* (Ferrari et al., 2008). PG plants do not show constitutive expression of typical marker genes for salicylic- (SA-), ethylene-, or JA-dependent defense responses (Raggi et al., 2015) but accumulate high levels of ROS and peroxidase activity in their tissues (Ferrari et al., 2008). Accumulation of ROS in PG plants is associated to an increased expression of the class III peroxidase gene *AtPRX71* (Raggi et al., 2015), whose overexpression was previously shown to confer resistance to *B. cinerea* (Chassot et al., 2007). Robust resistance to *B. cinerea* was also previously reported in the *quasimodo2-1* (*qua2-1*, henceforth *qua2*) mutant, defective in the *QUASIMODO2*/*TUMOROUS SHOOT DEVELOPMENT2* (QUA2/TSD2) gene, which encodes a putative pectin methyltransferase required for the biosynthesis of de-esterified HG (Mouille et al., 2007; Verger, 2014). Notably, like PG plants, *qua2* shows upregulation of *AtPRX71* expression and accumulates high peroxidase activity and ROS levels (Raggi et al., 2015). Loss of *AtPRX71* partially suppresses the accumulation of ROS and the defect in cell expansion of *qua2*, whereas its overexpression in the WT results in high levels of ROS and reduced cell expansion (Raggi et al., 2015), indicating that AtPRX71-mediated ROS production contributes to the growth defects observed in *qua2*, whereas its role in the resistance to *B. cinerea* of this mutant is unknown. In this work, we show that PG and *qua2* plants, but not other unrelated CW mutants, display increased leaf cuticle permeability and that exogenous ABA suppresses both cuticle permeability and *Botrytis* resistance of these plants. Notably, loss of *AtPRX71* restores cuticle functionality and fungal susceptibility in *qua2*, suggesting that a peroxidase-mediated increase in cuticle permeability is a major determinant of the robust resistance to *B. cinerea* observed in plants with a defective HG.

## Materials and Methods

### Plant Material and Growth Conditions

All *Arabidopsis thaliana* plants used in this work belong to Columbia (Col-0) ecotype. The following mutants were obtained from the Nottingham *Arabidopsis* Stock Center: *prc1-1* (Desnos et al., 1996), *ixr1-2* (Scheible et al., 2001), *mur1-1* (Reiter et al., 1993), *mur4-1* (Reiter et al., 1997), and *fer-4* (Duan et al., 2010). Isolation of *atprx71-1* and the *qua2-1 atprx71-1* double mutant was previously described (Raggi et al., 2015), as well as generation of transgenic plants overexpressing the *A. niger pga* gene (PG plants; Lionetti et al., 2010) or *AtPRX71* (35S::PRX71 plants; Raggi et al., 2015). Seeds of *qua2* (Mouille et al., 2007) and *tsd2-1* (Krupková et al., 2007) were kind gifts of Gregory Mouille (Institut National de la Recherche Agronomique, Versailles, France) and Thomas Schmülling (Freie Universität Berlin, Berlin, Germany), respectively. Plants were grown in a growth chamber with a light intensity of 110–120μmolm^−2^ s^−1^ with a 12h/12h (dark/light) photoperiod, 70–75% humidity, and 20–22°C temperature. Before sowing, seeds were surface sterilized and stratified for 3days in the dark at 4°C. ABA treatments on adult plants were performed spraying rosette leaves of five-week-old plants with a solution of 100μm ABA (Duchefa) dissolved in ethanol [final ethanol concentration: 0.1% (v/v)].

### Fungal Infections

*Botrytis cinerea* growth and inoculation were performed as previously described (Ferrari et al., 2007; Galletti et al., 2008). Briefly, 5μl droplets of a spore suspension (5×10^5^ conidia ml^−1^) in 24g L^−1^ potato dextrose broth were inoculated on rosette leaves of five-week-old plants grown in soil (two droplets per leaf, three or four fully expanded leaves per plant). Inoculated plants were covered with a transparent plastic dome to maintain high humidity and returned to the growth chamber. Lesions were photographed at 3days post-infection and their area was measured using ImageJ software.^1^

### Germination and Pathogenicity Assays in the Presence of Leaf Diffusates

Leaf diffusates were isolated as previously described (Bessire et al., 2007) with minor modifications. Briefly, potato dextrose broth (Duchefa) droplets (5μl) were placed on the adaxial face of rosette leaves of five-week-old plants in 100% humidity. After 44h of incubation, the diffusates were recovered and inoculated with *B. cinerea* spores to a final concentration of 5×10^5^ml^−1^. *In vitro* germination was calculated after 8h of incubation. *In vivo* infections were performed using the same mixtures of leaf diffusates and spore, and lesion area was measured at 72h after infection.

### Gene Expression Analysis

To analyze gene expression, leaves were frozen in liquid nitrogen and homogenized with an MM301 Ball Mill (Retsch) for about 1min at 25Hz. Total RNA was extracted with EUROGOLD TriFast-Nucleic Acids Isolation Reagent according to the manufacturer’s instructions. 1μg of total RNA was retrotranscribed into cDNA with Improm II Reverse Transcriptase (Promega). cDNA mixed with iTaq Universal SYBR Green Supermix (Bio-Rad) and primers specific for genes of interest was loaded on a 96-well plate and run on CFX96 Real-time System (Bio-Rad). Gene expression, relative to *UBIQUITIN5* (*UBQ5*), was calculated according to the ΔΔCT method (Livak and Schmittgen, 2001). Primer sequences were the following: *UBQ5* (At3g62250), GGAAGAAGAAGACTTACACC and AGTCCACACTTACCACAGTA; *PAD3* (At3g26830), TCGCTGGCATAACACTATGG and TTGGGAGCAAGAGTGGAGT; *PR-1* (At2g14610), GGGAAAACTTAGCCTGGGGT and GCACATCCGAGTCTCACTGA; *PDF1.2* (At5g44420), TCTCTTTGCTGCTTTCGACG and ACTTGTGTGCTGGGAAGACA; *MYB96* (At5g62470), TGCTATGGCTGCCCATCTGTT and AGCTGCAGATTGAGATGGACTA; *CER1* (At1g02205), TCCACTCCTGTGAGAACTGGT and TACTTGGTCCAAATCCGAGAGA; *LTP3* (At5g59320), TGCGAAGAGCATTTCTGGTCTC and TGATGTTGTTGCAGTTAGTGCTC; *LTP4* (At5g59310), AGTCCGCTGCAAAAGGGGTTA and TTGATGGTGGCGCAGTTGGT; *KCS2* (At1g04220), GCTAAACAGCTTCTTCAGGTTCA and TCGGAAGATGCAGTTTGAGAGA; *BDG1* (At1g64670), AGAAACAGGATGCGAACGTACT and ACATGGTCCAAATAAGCCTCTAC; and *LACS2* (At1g49430), GTAGAGGAGTTCTTGAGAATCATT and AACTCTCAGTCAATCCATAACCTT. The primers utilized for the amplification of the *B. cinerea BcAct* actin gene (accession no. AJ000335) were the following: AAGTGTGATGTTGATGTCC and CTGTTGGAAAGTAGACAAAG (Ferrari et al., 2008).

### Cuticle Permeability Assays

Toluidine blue staining was performed as previously described (Tanaka et al., 2004) with minor modifications. 5μl droplets of 0.05% (w/v) toluidine blue (Sigma-Aldrich) solution were incubated for 2h on adaxial surface of fully expanded leaves from 35-day-old plants. After incubation, leaves were washed and photographed.

Chlorophyll extraction and quantification were performed as described previously (Sieber et al., 2000). Leaves were detached, weighed, and immersed in 10ml of 80% (v/v) ethanol. Chlorophyll extraction occurred in the dark at room temperature with gentle shaking. Aliquots were taken at indicated time after immersion. Total chlorophyll content was quantified by measuring absorbance at 647 and 664nm, and micromolar concentration of chlorophyll per gram of fresh weight was calculated with the following equation: [19.536×(Abs 647nm)+7.936×(Abs 664nm)] g^−1^ (Sieber et al., 2000).

### ROS and Cell Death Detection

ROS accumulation was revealed by diaminobenzidine tetrahydrochloride (DAB, Sigma-Aldrich) staining as described previously (Daudi and O’Brien, 2012). Briefly, detached leaves were vacuum infiltrated with 0.1% (w/v) DAB in 10mm Na_2_HPO_4_ for 5min and incubated for 8h in the dark under gentle shaking. Chlorophyll was removed using a 3:1:1 ethanol:acetic acid:glycerol solution at 90–95°C for 15min. Leaves were rinsed in distilled water and photographed.

Cell death was estimated with Evans blue staining as previously described (Dong et al., 2007) with minor modifications. Briefly, detached rosette leaves were vacuum infiltrated for 5min with a 0.1% Evans blue (w/v) solution and stained at room temperature for 3–4h. Samples were washed twice with phosphate buffer and chlorophyll was extracted overnight with 80% ethanol.

### Microscopy

For gap area determination, leaves of five-week-old soil grown plants were harvested and cleared with 80% ethanol overnight. Leaves were stained with ruthenium red (0.05% w/v) for 10–60min at room temperature and washed with water. Images were taken with a Nikon Digital Sight DS-Fi1c Camera and examined using a Nikon Eclipse E200 Microscope. Gap area was measured using ImageJ.^2^ At least two leaves from each of four individual plants per genotype were analyzed.

Transmission electron microscopy (TEM) experiments were performed largely as previously described (Nafisi et al., 2015b). Leaf pieces (approximately 2×4mm) were cut out from the middle part of mature rosette leaves, avoiding the larger veins. The samples were fixed for 2h under vacuum with Karnovsky’s fixative (2.5% v/v glutaraldehyde and 2% w/v formaldehyde in 0.1M sodium cacodylate buffer, pH 7.2; all from Sigma-Aldrich). The fixative was replaced by a 0.1M sodium cacodylate buffer, pH 7.2, and samples placed in a tissue rotator for 20min, then washed with 0.1M sodium cacodylate buffer, and incubated for additional 20min. The buffer was removed and replaced by osmium fixative (1% w/v osmium tetroxide in 0.1M sodium cacodylate buffer, pH 7.2; Sigma-Aldrich). The samples were left for post-fixation for 1h in the rotator and thereafter rinsed in 0.1M sodium cacodylate buffer and water for 20min, respectively. Dehydration was performed by a series of 50 to 100% v/v acetone solutions, which were replaced every 20–30min. Spurr’s resin (Polysciences) was then added in a 1:3, 1:1, and 3:1 ratio with acetone and rotated for 30min in each step. Pure Spurr’s resin was applied, and samples were left without lids for 1h so that any remaining acetone could vaporize. The samples were left overnight, and the resin was changed once before embedding in flat molds in the oven at 70°C for 8h. An EM UC7 Ultramicrotome (Leica Microsystems) was used for making ultrathin sections of 40nm thickness with a diamond knife. Sections were captured on carbon-coated grids, contrast stained with a 1% w/v uranyl acetate solution and a 0.5% w/v lead citrate solution, washed, and dried into filter paper. The images were taken with a CM100 Transmission Electron Microscope (Philips) with a side-mounted 11 MP camera (Morada) set to an acceleration voltage of 80kV. Quantification of cuticle thickness was performed with ImageJ. A total of 75 individual measurements were made for the upper leaf epidermis in each genotype at the magnification of 64,000×.

### Chemical Analysis of Cutin

For the chemical analysis of cutin, five-week-old rosettes were extracted in isopropanol/0.01% butylated hydroxytoluene and then delipidized twice (for 16h and 8h) with each of the following solvents: chloroform:methanol (2:1), chloroform:methanol (1:1), and methanol (with 0.01% butylated hydroxytoluene), under agitation, before being dried for 3days under vacuum. Depolymerization was performed by base catalysis (Li-Beisson et al., 2013). In brief, dried plant samples were transesterified in 2ml of reaction medium. 20ml of reaction medium was composed of 3ml methyl acetate, 5ml of 25% sodium methoxide in dry methanol, and 12ml dry methanol. 2mg of methyl heptadecanoate and 4mg of ω-pentadecalactone were added per sample as internal standards. After incubation of the samples at 60°C for 2h, 3.5ml dichloromethane, 0.7ml glacial acetic acid, and 1ml 0.9% NaCl (w/v) Tris 100mM (pH 8.0) were added to each sample and subsequently vortexed for 20s. After centrifugation (1,500*g* for 2min), the organic phase was collected, washed with 2ml of 0.9% NaCl, and dried over sodium sulfate. The organic phase was then recovered and concentrated under a stream of nitrogen. The resulting cutin monomers were acetylated at 70°C for 2h and injected out of hexane on a HP-5 MS column (J&W Scientific) in a gas chromatograph coupled to a mass spectrometer and a flame ionization detector (Agilent 6890 N GC Network systems). The temperature cycle of the oven was the following: 2min at 50°C, increment of 20°Cmin^−1^ to 160°C, of 2°Cmin^−1^ to 250°C and 10°Cmin^−1^ to 310°C, and held for 15min. Two independent experiments were performed with four replicates for each genotype and treatment, respectively, and a representative dataset is presented.

## Results

### *Arabidopsis* Plants With Altered HG Display a Robust Resistance to *B. Cinerea* and Enhanced Cuticle Permeability That Are Both Suppressed by Exogenous ABA

**Figure 1:**
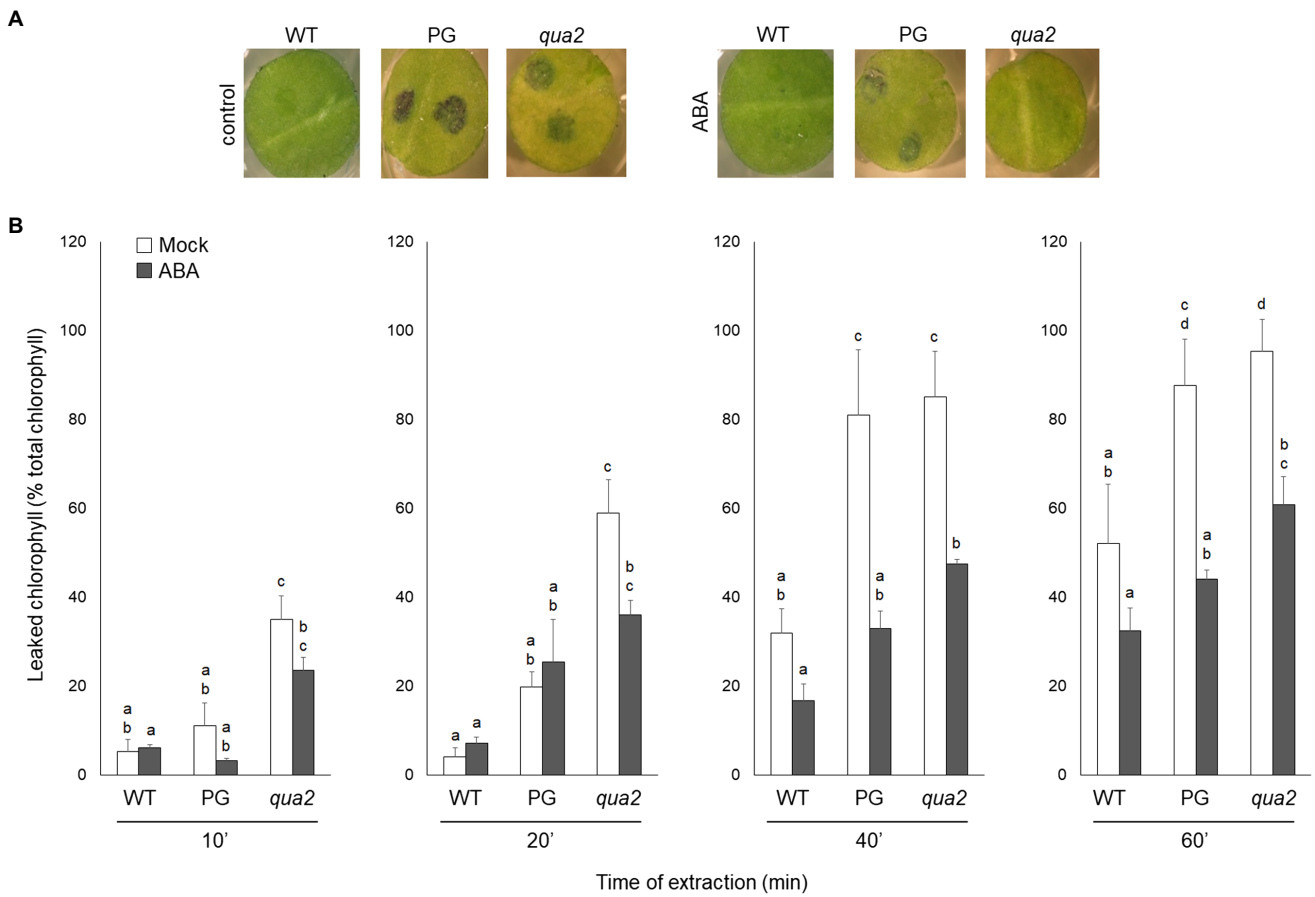
Plants with altered HG have increased leaf cuticle permeability that is suppressed by exogenous ABA. **(A,B)**, WT, PG, and *qua2* five-week-old plants were sprayed with 0.01% MeOH or 100μm ABA and, after 24h, rosette leaves were collected. **(A)**, Leaf disks were stained with droplets of toluidine blue and washed with water. **(B)**, Leaves were incubated for the indicated times with 80% EtOH and the percentage of total chlorophyll released in the medium was spectrophotometrically measured. Bars indicate average percentage of total chlorophyll leaked at the indicated time of incubation ± *SD* (*n* = 3). For each time point, letters indicate statistically significant differences, according to two-way ANOVA followed by Tukey’s significance test (*p* < 0.05). These experiments were repeated three times with similar results.

**Figure 2:**
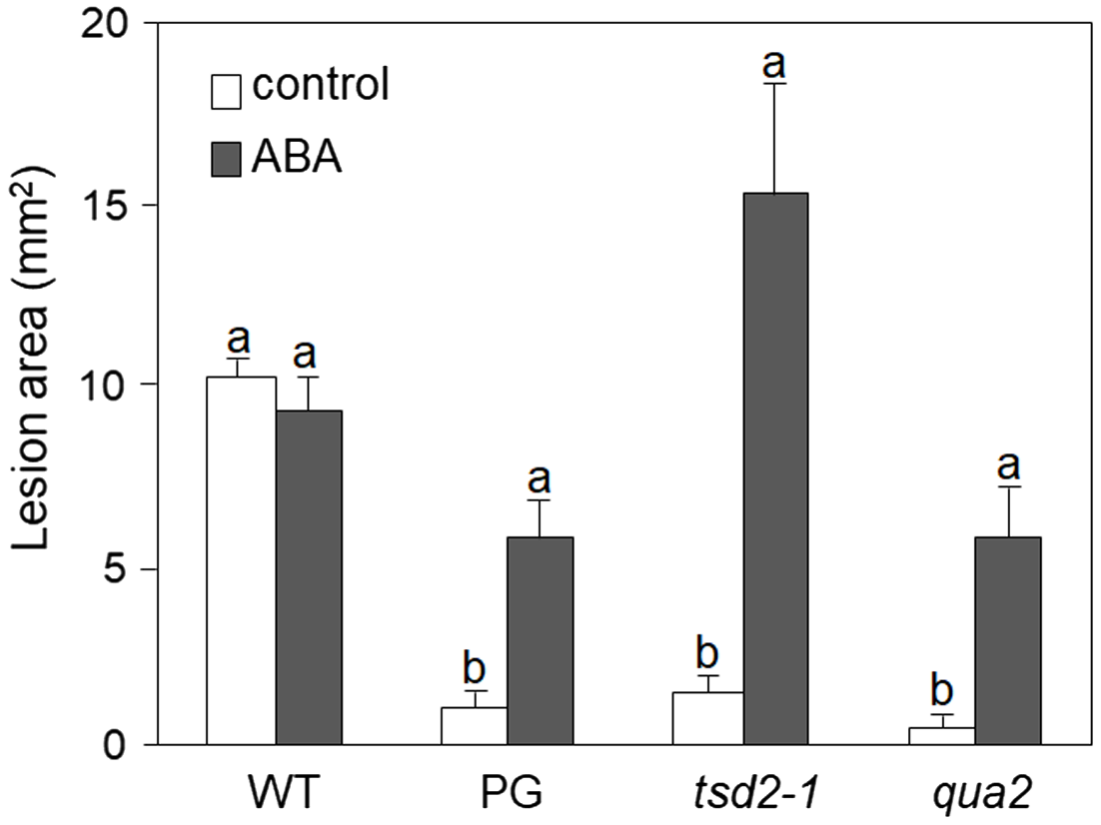
Resistance to *Botrytis cinerea* conferred by altered HG is suppressed by exogenous ABA. Rosette leaves of five-week-old WT, PG, *qua2*, and *tsd2-1* plants were treated with 0.01% MeOH (white bars) or 100μm ABA (grey bars) and, after 24h, inoculated with a *B. cinerea* spore suspension. Lesion area was measured 2days after inoculation (*n* > 10 lesions for each genotype). Bars indicate average lesion area ± *SE*. Different letters indicate statistically significant differences, according to two-way ANOVA followed by Tukey’s significance test (*p* < 0.05). This experiment was repeated three times with similar results.

It was previously reported that PG and *qua2* plants display a robust resistance to *B. cinerea* (Ferrari et al., 2008; Verger, 2014), that resembles that of *Arabidopsis* plants with altered cuticle (Bessire et al., 2007; Chassot et al., 2007; Tang et al., 2007). Moreover, ABA positively regulates cuticle structure and functionality (Curvers et al., 2010; Cui et al., 2016) while negatively affecting resistance to *B. cinerea* (Audenaert et al., 2002; Anderson et al., 2004; Asselbergh et al., 2007; L’Haridon et al., 2011). We therefore examined whether plants with HG defects show changes in cuticle permeability, and whether ABA affects this phenotype. Rosette leaves of WT, PG, and *qua2* plants were sprayed with a control solution or with ABA, and cuticle permeability was evaluated after 24h using two different assays: toluidine blue staining of the leaf surface (Figure 1A) and measurement of chlorophyll leakage in ethanol (Figure 1B). The results of both assays clearly indicated that cuticle permeability is increased in PG and *qua2* plants, compared to the WT, and that pre-treatments with ABA significantly reduce permeability in both lines (Figures 1A,B). We then tested whether the strong resistant phenotype associated to HG alterations is also negatively affected by this hormone. PG plants and the two allelic mutants *qua2* and *tsd2-1* (Krupková et al., 2007) were sprayed with ABA and, after 24h, inoculated with *B. cinerea*. Control-treated PG and mutant plants displayed, as expected, a strong resistance to the fungus (Figure 2). ABA pre-treatments did not significantly alter basal susceptibility in WT plants but restored WT-like levels of susceptibility in all transgenic and mutant lines (Figure 2). These data suggest that an increased cuticle permeability might be responsible for the enhanced resistance against *B. cinerea* caused by defects in HG composition. Since transgene expression in PG plants was observed to vary among generations, all subsequent experiments were conducted on *qua2* plants, which showed similar phenotypes in terms of resistance to *B. cinerea*, leaf permeability, and response to ABA.

Previous work indicates that on the surface of *Arabidopsis* leaves having an increased cuticle permeability antifungal compounds effective against *B. cinerea* are present, possibly contributing to their robust resistance to this pathogen (Bessire et al., 2007). Consistently, leaf diffusates from *qua2* plants reduced *in vitro B. cinerea* spore germination (Supplementary Figure 1A) and suppressed *in vivo* disease development (Supplementary Figure 1B). These results support the conclusion that the increased cuticle permeability of plants with altered HG has a major role in their enhanced resistance to *B. cinerea*.

**Figure 3:**
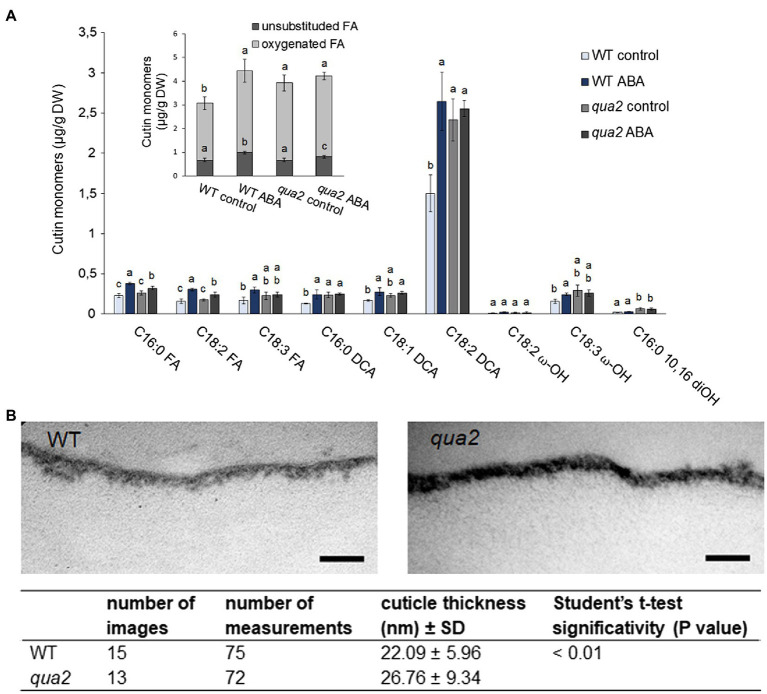
Cutin deposition and cuticle thickness are enhanced in *qua2* mutant leaves. **(A)**, Five-week-old plants were sprayed with 0.01% MeOH (control) or 100μm ABA and leaves collected after 24h. Quantification of transesterified aliphatic and aromatic cutin monomers reveals an increase of main cutin components in *qua2* which are not further increased after ABA treatment. The graph shows the analysis of the principal cutin monomers and the inset shows the sum of the indicated monomer classes per genotype and treatment. Data are mean ± *SD*; *n* = 4 replicates. Different letters indicate significant differences as determined by ANOVA followed by Tukey’s significance test (*p* < 0.05). DW, dry weight; DCA, dicarboxylic acid; ω-OH, ω-hydroxy acid; and FA, fatty acid. **(B)**, TEM images of the cuticle of epidermal cells of the adaxial face of rosette leaves of five-week-old WT (top panel) and *qua2* (middle panel) plants. Pictures are representative of 15 (WT) and 13 (*qua2*) images. Bars, 125nm. Bottom panel measurement of cuticle thickness from images taken as above described.

Because alterations of cuticle permeability may be due to a defective cutin, its composition was investigated. Compared to the WT, several oxygenated ester-bound lipid compounds typical for cutin were increased in untreated *qua2* mutant leaves, including the main cutin component, *i.e*., 18:2 dicarboxylic acid (18:2 DCA; Figure 3; Li-Beisson et al., 2013), leading to an overall increase of oxygenated cutin monomers by 36% (Figure 3 insert), while unsubstituted ester-bound fatty acids were unchanged. ABA treatment increased unsubstituted and oxygenated ester-bound lipid compounds in WT by 46.1 and 44.5%, respectively. ABA treatment also increased unsubstituted ester-bound lipids in the *qua2* mutant by 20.9%, while oxygenated cutin monomers were however not further increased (Figure 3A). In addition, TEM analysis did not reveal major defects in the ultrastructure of the cuticle in the epidermal cells of *qua2* rosette leaves (Figure 3B), except for a slight but significant increase in cuticle thickness which might be due to the increased cutin monomer levels.

We also evaluated the expression of genes involved in the biosynthesis of cutin and waxes in fully expanded rosette leaves of *qua2* plants. In particular, we measured transcript levels for the following genes: *BDG*, required for proper cutin biosynthesis (Kurdyukov et al., 2006); *LACS2*, involved in the biosynthesis of cutin and wax monomers (Schnurr et al., 2004; Lü et al., 2009); *ECERIFERUM1* (*CER1*) and *3-KETOACYL-COENZYME A SYNTHASE 2* (*KCS2*), both implicated in cuticular wax biosynthesis (Lee et al., 2009; Bourdenx et al., 2011; Bernard et al., 2012); and *MYB96*, encoding a transcription factor that positively regulates the expression of wax-related genes (Seo et al., 2011). We also evaluated the expression of *LIPID TRANSFER PROTEIN 3* (*LTP3*), a direct target of MYB96 (Guo et al., 2013), and its close homolog *LTP4*. This set of genes was selected because they all show altered expression in the *OG hypersensitive 1* (*ohy1*) mutant, that, like *qua2*, has a permeable cuticle, increased peroxidase levels and increased resistance to *B. cinerea* (Survila et al., 2016). Basal levels of expression of *LACS2* and *BDG* were not significantly different in WT and *qua2* rosette leaves, though they appeared more variable in the mutant (Supplementary Figure 2). Expression of *MYB96*, *LTP3*, and *LTP4* was significantly enhanced in *qua2*, compared to the WT, whereas *CER1* expression did not significantly change and *KCS2* expression was reduced in the mutant (Supplementary Figure 2). These data indicate that *qua2* does not show a major reduction of the expression of genes involved in cutin or wax biosynthesis that might explain its defect in permeability. Notably, ABA treatments induced the expression of all tested genes in both genotypes (Supplementary Figure 2), indicating that ABA-mediated regulation of genes involved in cuticle formation is not impaired in *qua2*.

### *AtPRX71* Contributes to Cuticle Permeability, Loss of Cell Adhesion, and Resistance to *B. Cinerea* in *qua2* Plants

**Figure 4:**
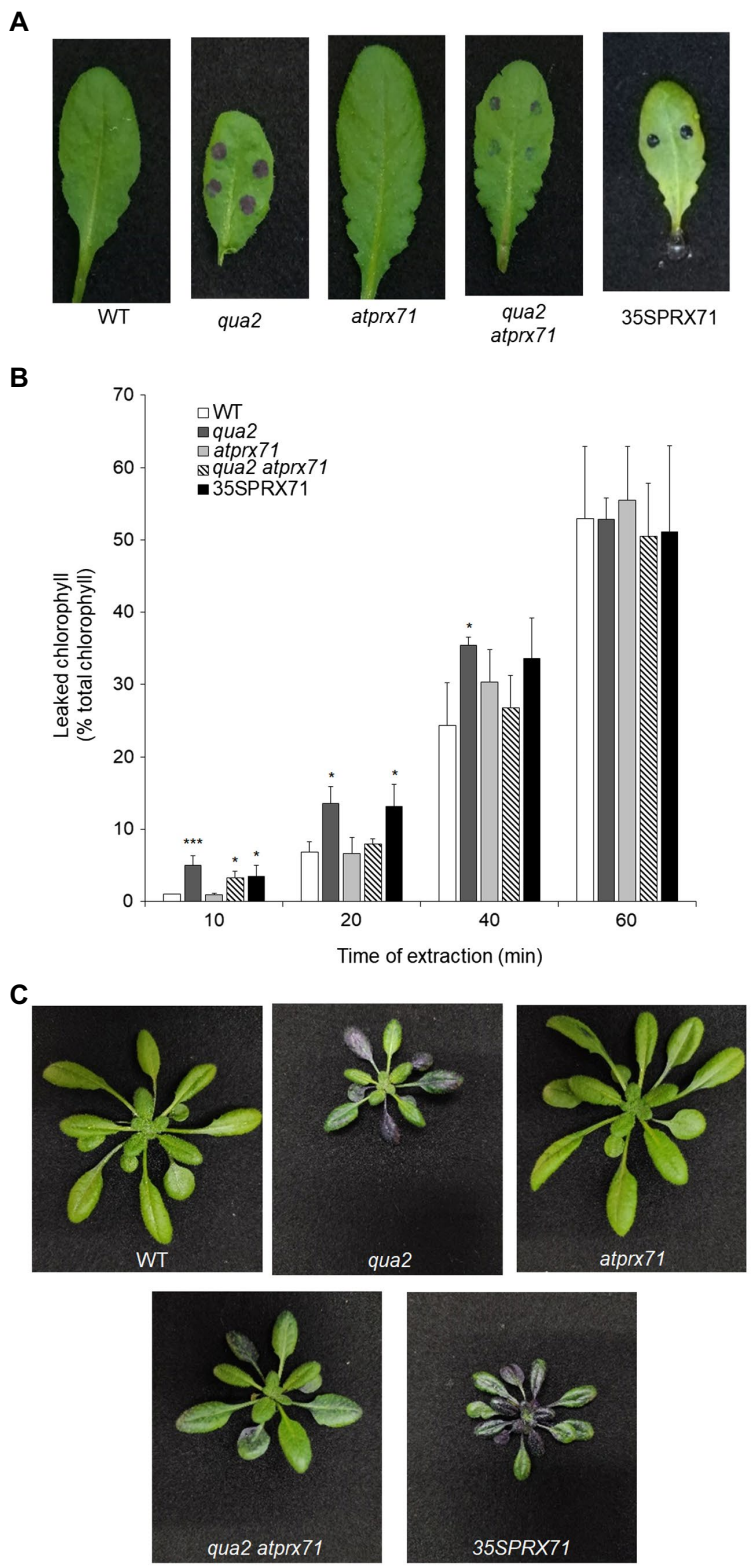
Increased cuticle permeability of *qua2* leaves is partially dependent on *AtPRX71*. Permeability of the cuticle of fully expanded rosette leaves **(A)** of five-week-old Col-0 (WT), *qua2*, *atprx71*, *qua2 atprx71*, and 35S::AtPRX71 #22 (35SPRX71) plants was determined by toluidine blue staining. **(B)** The amount of chlorophyll leached from rosette leaves as in **(A)** was measured after incubation in 80% EtOH at the indicated times. Bars indicate average concentration ± *SD* (*n* = 3). For each time point, asterisks indicate significant differences, according to Student’s t-test between WT and mutants (^*^p < 0.05; ^***^p < 0.01). These experiments were repeated three times with similar results. **(C)** Toluidine blue staining of the entire rosette of five-week-old plants. Representative images of at least five plants per genotype are shown.

Cuticle defects may lead to the expression of apoplastic peroxidases and accumulation of ROS (Chassot et al., 2007), as also observed in plants with defects in HG (Ferrari et al., 2008; Raggi et al., 2015). Moreover, overexpression of apoplastic peroxidases in WT plants also leads to ROS accumulation and enhanced cuticle permeability, responses that are both suppressed by ABA (Survila et al., 2016). It can be therefore hypothesized that the increased permeability of *qua2* might depend on its high levels of *AtPRX71* expression. To assess this, we analyzed cuticle permeability in fully expanded rosette leaves of WT, *qua2*, and *atprx71-1* (henceforth, *atprx71*) plants, in a *qua2 atprx71* double mutant, and in two transgenic lines overexpressing *AtPRX71* (*35S::PRX71* #22 and 24; Raggi et al., 2015). As expected, leaves of plants overexpressing *AtPRX71* showed greatly enhanced cuticle permeability, compared to untransformed plants, as determined by both toluidine blue staining and leaf chlorophyll leakage measurement (Figures 4A,B). Loss of *AtPRX71* did not affect cuticle permeability in the WT background but partially rescued the cuticle permeability defect of *qua2* (Figures 4A,B). Toluidine blue staining of whole rosettes showed that the cuticle of the entire lamina of mature (fully expanded) leaves of *qua2* plants is permeable, whereas younger leaves were not stained (Figure 4C). Mature leaves of the *qua2 atprx71* double mutant showed irregular patches of permeable cuticle, supporting the hypothesis that *AtPRX71* contributes to the increased cuticle permeability of *qua2* (Figure 4C). Plants overexpressing *AtPRX71* showed a very high cuticle permeability of the entire lamina of younger leaves, and an irregular distribution of permeable cuticle patches in older leaves (Figure 4C). Interestingly, ABA pre-treatments, which suppressed cuticle permeability in *qua2* (Figures 1A,B), also reduced ROS accumulation in this mutant, as determined by DAB staining (Supplementary Figure 3). These results suggest that accumulation of ROS mediated by *AtPRX71* is at least partially responsible for the increased cuticle permeability of *qua2* leaves. In addition, despite the high basal levels of ROS, no significant increase in Evans blue staining, indicative of cell death, was observed in *qua2* leaves, both in uninfected plants and at early stages of *B. cinerea* infection (16h post-infection, hpi), though staining at 40hpi was reduced in the mutant, possibly as a consequence of the reduced ability of the fungus to infect this mutant (Supplementary Figure 4A) and suggesting that, in *qua2*, the fungus is restricted at an early stage of infection. Consistently, expression of the fungal actin *BcAct* gene was comparable in WT and *qua2* plants at 24hpi but increased at 36hpi only in WT plants (Supplementary Figure 4B).

**Figure 5:**
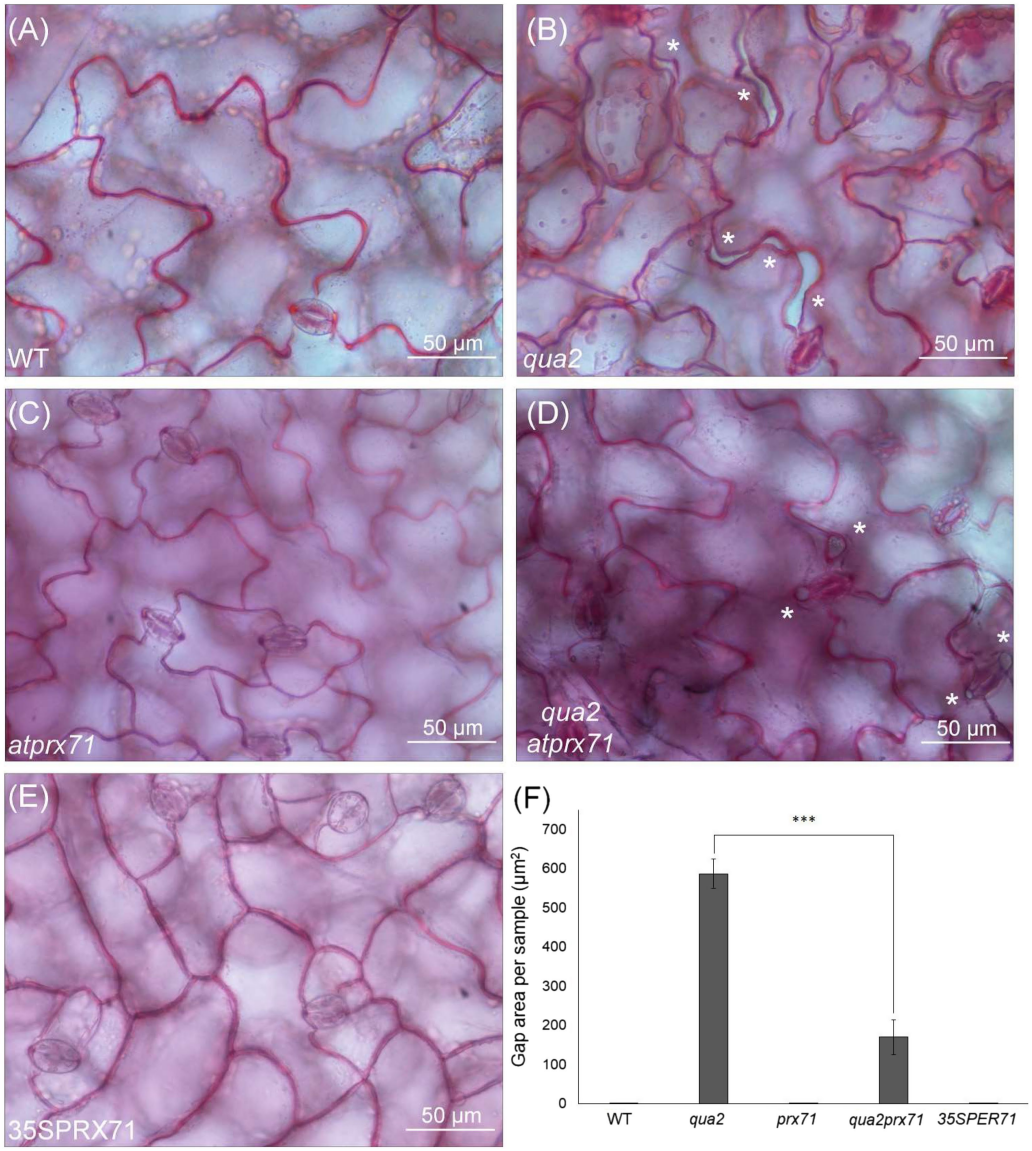
Cell-adhesion defects in *qua2* leaves are suppressed by loss of *AtPRX71*. Rosette leaves of five-week-old Col-0 (WT) **(A)**, *qua2* **(B)**, *atprx71* **(C)**, *qua2 atprx71* **(D)**, and 35**S**::AtPRX71 #22 (35SPRX71) **(E)** plants were stained with ruthenium red and epidermal cells of the adaxial face were photographed. Asterisks indicate gaps between adjacent cells. **(A–E)**, Representative images of at least five images per genotype. **(F)**, For each experiment, total area of the gaps between adjacent cells per image was analyzed (total area of images = 60,000μm^2^). Bars indicate average gap area ± *SD* (*n* = 3). Asterisks indicate statistically significant difference between *qua2* and *qua2 atprx71*, according to Student’s t-test (^***^p < 0.01).

It was previously shown that *QUA2* is required for normal cell adhesion (Mouille et al., 2007). Since *qua2* plants do not show major changes in cuticle ultrastructure and do not display a reduction in ester-bound lipids or in the expression of most cuticle-related genes that we have analyzed, we speculated that a reduced adhesion of the epidermal cells might contribute to its increased permeability. Indeed, cracks between adjacent cells and opening of the cuticle at cell junctions were previously described in the hypocotyl of *qua1-1* (Verger et al., 2018), a mutant for a glycosyltransferase that is required for pectin synthesis and cell adhesion (Bouton et al., 2002; Mouille et al., 2007). Indeed, examination of the surface of *qua2* rosette leaves revealed the presence of gaps between adjacent epidermal cells (Figure 5). Notably, the area of these gaps was significantly reduced in the *qua2 atprx71* double mutant (Figure 5), indicating that increased peroxidase levels in plants with altered HG exacerbate the adhesion defect in *qua2* leaf epidermis. In contrast, overexpression of *AtPRX71* in a WT background did not result in any detectable loss of cell adhesion (Figure 5). Moreover, ABA treatments did not affect epidermal cell gap area in *qua2* (Supplementary Figure 5). Interestingly, a mutation in the *ESMERALDA1* gene, previously shown to suppress the cell adhesion defect in *qua2* without affecting its galacturonic acid content (Verger et al., 2016), also suppressed its increased cuticle permeability (Supplementary Figure 6), supporting the hypothesis that the defects in cell adhesion and in cuticle functionality in *qua2* are linked.

**Figure 6:**
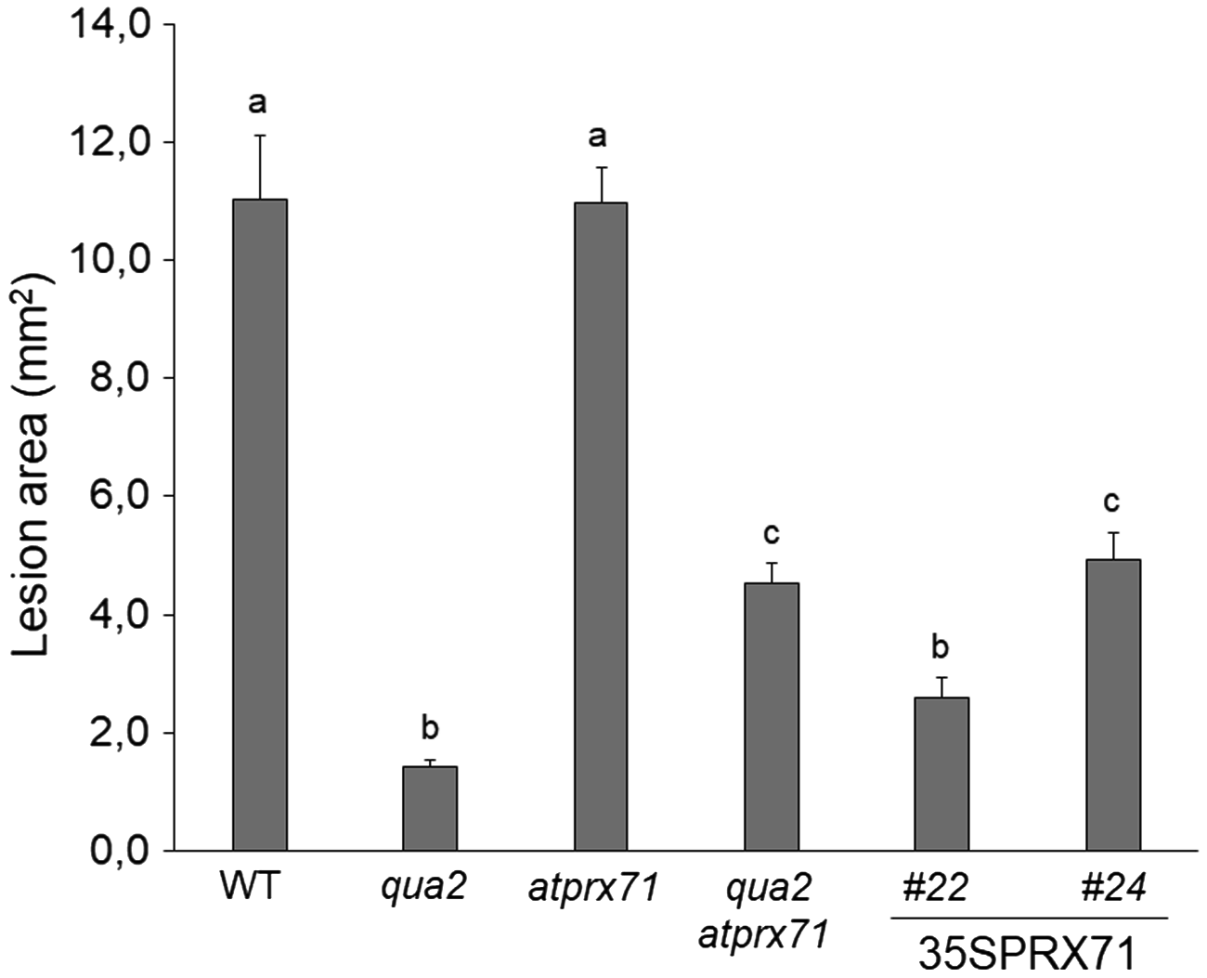
Resistance to *B. cinerea* in *qua2* is partially dependent on *AtPRX71*. Rosette leaves of five-week-old WT plants, *qua2, atprx71*, and *qua2 atprx71* plants, and of two independent transgenic lines overexpressing *AtPRX71* (35SPRX71 #22 and #24) were inoculated with a *B. cinerea* spore suspension, and lesion area was measured 2days after inoculation (*n* > 10 for each genotype). Bars indicate average lesion area ± *SE*. Different letters indicate statistically significant differences, according to one-way ANOVA followed by Tukey’s significance test (*p* < 0.01).

We next evaluated whether *AtPRX71* contributes to the resistance to *B. cinerea* observed in *qua2*. As previously reported (Raggi et al., 2015), symptoms caused by this pathogen in the *atprx71* mutant were comparable to those observed in the WT (Figure 6), confirming that *AtPRX71* is not necessary for basal resistance to this fungus in plants with normal pectin composition. *AtPRX71* overexpression was sufficient to significantly increase resistance to *B. cinerea* (Figure 6), a result also in agreement with previous observations (Chassot et al., 2007). Notably, the *qua2 atprx71* double mutant was significantly less resistant than *qua2*, though it was still more resistant than the WT (Figure 6). Taken together, these results suggest that *AtPRX71* is required for the strong resistance to *B. cinerea* of *qua2*, possibly because it contributes to increased cuticle permeability.

To further investigate the role of *AtPRX71* in *qua2* resistance, we examined the expression of three defense-related genes, *PHYTOALEXIN DEFICIENT 3* (*PAD3*), *PATHOGENESIS-RELATED 1* (*PR-1*), and *PLANT DEFENSIN 1.2* (*PDF1.2*), during fungal infection of WT, *qua2*, *atprx71*, and *qua2 atprx71* plants. These genes were selected since they are markers for different defense-related pathways: *PR-1* and *PDF1.2* are well-known markers for the activation of SA-dependent and JA/ethylene-dependent responses, respectively (Glazebrook, 2005), whereas *PAD3*, encoding a cytochrome P450 required for the biosynthesis of camalexin (Schuhegger et al., 2006), is rapidly induced by *B. cinerea* and by DAMPs and MAMPs independently of SA, JA, and ethylene (Ferrari et al., 2007; Gravino et al., 2015) and is required for basal and elicitor-induced resistance to this fungus (Ferrari et al., 2003, 2007). At 8hpi, *PAD3* transcript levels were significantly higher in *qua2* than in the WT (Supplementary Figure 7A), suggesting that plants with altered HG display an anticipated response to the pathogen. This early induction of *PAD3* expression also occurred in the *qua2 atprx71* double mutant (Supplementary Figure 7A), indicating that it does not depend on an increased peroxidase activity. At 24hpi, levels of *PAD3* transcripts increased to a greater extent in the WT than in *qua2* and *qua2 atprx71* plants (Supplementary Figure 7A), possibly because fungal growth was reduced in both mutants. Expression of *PR-1* and *PDF1.2* during *B. cinerea* infection increased at later time points than *PAD3* and was similar in WT and *qua2* plants (Supplementary Figures 7B,C), suggesting that activation of SA- and JA/ET-mediated pathways does not significantly contribute to the resistance observed in the mutant.

### Cuticle Permeability and Resistance to *B. Cinerea* in Mutants Affected in Different Primary CW Components

**Figure 7:**
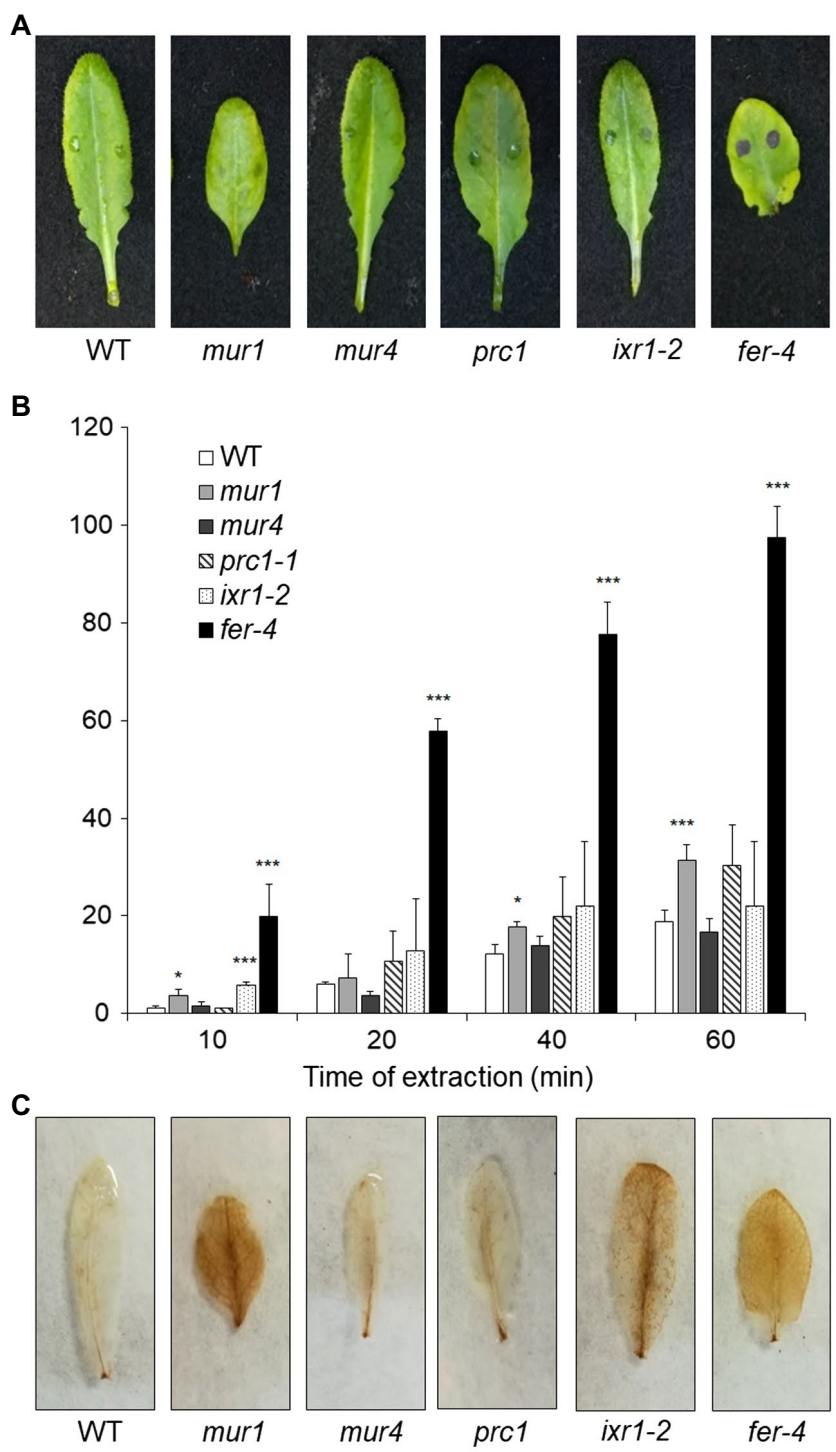
Cuticle permeability and ROS accumulation in mutants with impaired cell wall integrity. **(A,B)**, Rosette leaves of five-week-old Col-0 (WT), *mur1*, *mur4*, *prc1*, *irx1-2*, and *fer-4* plants were stained with toluidine blue **(A)** to assay cuticle permeability or **(B)** incubated for the indicated times with 80% EtOH, and the concentration of chlorophyll leaked in the medium and of total chlorophyll was spectrophotometrically measured. Bars in **(B)** indicate average percentage of total chlorophyll leaked at the indicated time of incubation ± *SD* (*n* = 3). For each time point, asterisks indicate statistically significant differences with the control-treated WT samples, according to Student’s t-test (^***^p < 0.01; ^*^p < 0.01). **(C)**, rosette leaves as in **(A,B)** were stained with DAB for ROS accumulation. These experiments were repeated three times with similar results.

Since plants with altered HG show increased ROS accumulation, cuticle permeability and resistance to *B. cinerea*, we assessed whether mutants with defects in other CW polysaccharides also display these phenotypes. We tested *prc1* and *ixr1-2*, which have mutations in *CESA1* and *CESA3*, respectively (Fagard et al., 2000; Scheible et al., 2001), required for primary CW cellulose deposition (Fagard et al., 2000; Scheible et al., 2001; Desprez et al., 2007; Persson et al., 2007), *murus1* (*mur1*), defective in a GDP-Man-4,6-dehydratase required for the synthesis of Fuc and therefore impaired in xyloglucan and RGII integrity (O’Neill et al., 2001), and *murus4* (*mur4*), defective in a UDP-D-Xylose epimerase required for Ara biosynthesis (Burget et al., 2003) and characterized by a 50% reduction in Ara, mainly representative of RG-I and arabinogalactan proteins (AGPs; Reiter et al., 1997; Burget and Reiter, 1999). In addition, we analyzed the *fer-4* mutant (Duan et al., 2010), which is defective in the *FER* gene, important for pectin integrity perception (Li et al., 2016; Feng et al., 2018). Cuticle permeability was unaffected in *prc1*, *mur4*, and *ixr1-2* leaves, and only moderately increased in *mur1*, but was extremely increased in *fer-4* (Figures 7A,B), suggesting that cuticle defects are caused by alterations of HG integrity and/or of its perception, but not by alterations of other primary CW components. DAB staining revealed accumulation of ROS in *mur1*, *ixr1-2*, and *fer-4* rosette leaves (Figure 7C), indicating that high ROS levels *per se* are not sufficient to impair cuticle functionality. Whole rosette toluidine blue staining showed that all leaves of *prc1*, *mur4*, and *ixr1-2* plants have normal cuticle permeability, whereas patches of increased permeability could be observed in the older leaves of *mur1* plants (Supplementary Figure 8). The pattern of cuticle permeability of *fer-4* and *qua2* was similar, with almost complete staining of the lamina of fully expanded leaves, but not of younger leaves (Supplementary Figure 8).

**Figure 8:**
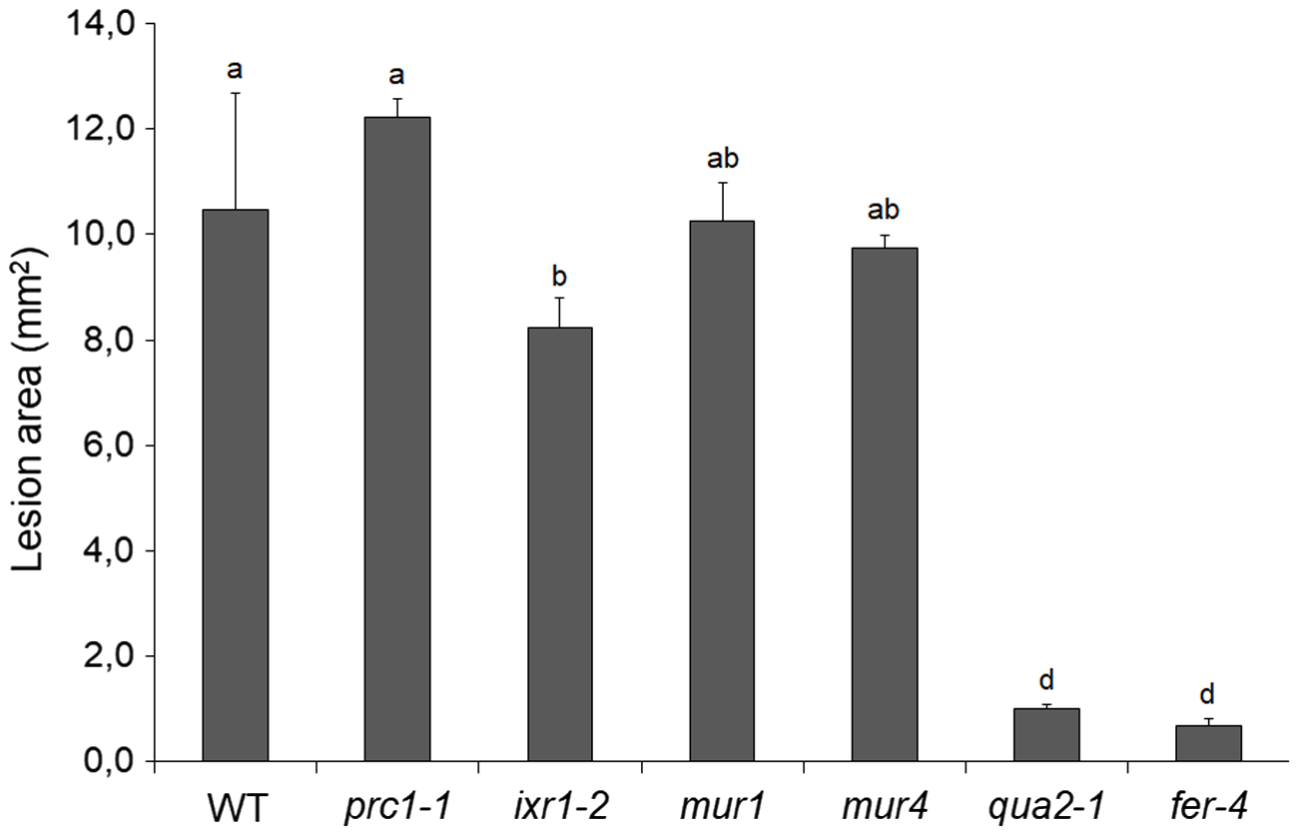
Resistance to *B. cinerea* in cell wall mutants. Rosette leaves of five-week-old WT plants and of *prc1*, *ixr1-2*, *mur1*, *mur4*, *qua2*, and *fer-4* mutant plants were inoculated with a *B. cinerea* spore suspension, and lesion area was measured 2days after inoculation (*n* > 12 lesions for each genotype). Bars indicate average lesion area ± *SE*. Different letters indicate statistically significant differences, according to one-way ANOVA followed by Tukey’s significance test (*p* < 0.01).

To test how changes in different primary CW polysaccharides affect susceptibility to *B. cinerea*, *prc1*, *ixr-2*, *mur1*, *mur4*, and *fer-4* plants were inoculated with the fungus and lesion area was measured after 48h. Only *fer-4* showed an almost complete resistance, comparable to that of *qua2*, whereas *ixr1-2* showed slightly reduced lesion size (Figure 8), consistent with the moderate resistance previously observed in the allelic mutant *ixr1-1* (Hernández-Blanco et al., 2007). Fungal resistance in all the other tested mutants was not significantly altered (Figure 8). Overall, our results suggest that defects in HG composition and/or integrity maintenance specifically confer a robust resistance to *B. cinerea* that correlates with loss of cell adhesion and enhanced cuticle permeability.

## Discussion

### Increased Cuticle Permeability Caused by Loss of HG Integrity Depends on AtPRX71 and Correlates With a Defective Epidermal Cell Adhesion

The traditional view of a cuticle separated from the underlying CW of epidermal cells is currently challenged by increasing evidence indicating that CW polysaccharides are entangled with cutin in the “cuticular layer” beneath the “cuticle proper” and that these polysaccharides affect cuticle deposition and functionality (Fernández et al., 2016). Indeed, some authors now prefer to consider the cuticle as an extreme modification of the outer CW (Domínguez et al., 2011), or a region of the CW extremely rich in lipids (Fernández et al., 2016). Here, we show that plants with defective HG, either caused by the *qua2* mutation or by expression of a fungal PG, and plants lacking a functional FER, supposed to act as a sensor of HG integrity (Lin et al., 2018), show increased cuticle permeability (Figures 1 and 7), supporting a strict interconnection between pectin composition and cuticle functionality. Indeed, pectin can be found in epidermal cells at the interface of the CW with the cuticle (Orgell, 1955; Jeffree, 2006), and the observation that the tomato mutant *sitiens*, defective in ABA biosynthesis, shows both altered cuticle and pectin composition (Curvers et al., 2010) further supports a link between these two structures. Overexpression of the *Arabidopsis* PME inhibitor PMEI5 leads to reduced permeability and organ fusion (Müller et al., 2013), suggesting that the degree of methylation of HG might be important for a functional cuticle. However, since *qua2* has a normal ratio between methylated and non-methylated HG (Mouille et al., 2007), total HG levels are probably more important in determining the defects in cuticle functionality of this mutant. It was previously reported that *qua2* is specifically affected in HG, without observable changes in other polysaccharides, including RG-I (Mouille et al., 2007). The recent observation that disrupted cellulose biosynthesis and orientation occurs in *qua2* mutants strongly indicates that an intact HG is required for proper CESA functionality (Du et al., 2020). However, it is unlikely that the reduced cuticle permeability observed in *qua2* and PG plants is due to a defect in the cellulose network, since mutants impaired in primary CW cellulose deposition (*prc1* and *ixr1-2*) do not show major changes in cuticle permeability.

The mechanism(s) linking altered HG integrity to loss of cuticle functionality is not clear, but our data suggest that production of ROS mediated by apoplastic peroxidases might play a role. We have previously found that *qua2*, as well as PG plants, accumulates high levels of ROS as a consequence of an increased expression of *AtPRX71* (Raggi et al., 2015). We show here that cuticle functionality is lost in plants overexpressing *AtPRX71*, and a mutation in this gene partially complements cuticle permeability in *qua2* (Figure 4). This is consistent with the previous observation that the *Arabidopsis ohy1* mutant, overexpressing another peroxidase gene, *PER57*, displays increased cuticle permeability (Survila et al., 2016). The increased permeability of both *qua2* and *ohy1* cuticles is therefore likely mediated by peroxidase-generated ROS, as supported by the observation that ABA suppresses both ROS accumulation and cuticle permeability in *ohy1* plants (Survila et al., 2016) as well as in plants with altered HG (Figure 1 and Supplementary Figure 3). How elevated peroxidase levels might influence cuticle functionality is still unclear. It was reported that expression of genes required for cutin biosynthesis is suppressed in the *ohy1* mutant (Survila et al., 2016), suggesting that elevated ROS produced by peroxidases negatively influence cutin production. Paradoxically, compared to the WT, *qua2* leaves display increased levels of ester-bound lipids, including 18:2 DCA, the main cutin component (Li-Beisson et al., 2013; Figure 3A), and a slight increase in cuticle thickness (Figure 3B), which might depend on increased cutin levels. Moreover, in contrast to *ohy1*, *qua2* plants do not display a reduction in the expression of *BDG* and indeed show increased basal transcript levels for *LACS2* (Supplementary Figure 2), consistent with the observed increase in residue-bound lipid content. An increased cutin monomer load was also previously observed in other mutants with altered permeability, namely, *lcr*, *fiddlehead* (*fdh*), *bdg*, *fatty acid desaturase2* (*fad2*), and the *fad3 fad7 fad8* triple (*fad* triple) mutant (Kurdyukov et al., 2006; Voisin et al., 2009; Dubey et al., 2020) and is suggestive of a compensatory response, possibly as a consequence of a more unstructured cutin and/or defects in wax-cutin associations, leading to the incorporation of more cutin-like material in the epidermal CWs of the mutant. Notably, *qua2* plants also display greatly increased basal levels of transcript levels of *MYB96, LTP3*, and its close homolog *LTP4* (Supplementary Figure 2). MYB96 is a transcription factor positively regulating genes involved in wax biosynthesis, and expression of *LTP3* was also previously shown to be upregulated in an activation-tagged *myb96-1D* mutant (Seo et al., 2011). These results suggest that the altered permeability caused by defects in HG is not due to a reduced expression of cuticle-related genes, at least in mature rosette leaves. Moreover, induction of the expression of cuticle-related genes by exogenous ABA was unaffected in *qua2* plants, ruling out a defect in ABA perception/signaling in this mutant.

The different expression pattern of cuticle-related genes in *ohy1* and *qua2* suggests that the impact of high peroxidase levels *per se* on cuticle formation is not completely overlapping with that of altered pectin composition, though they might contribute to increase cuticle permeability in the context of a defective pectin composition. Indeed, our results indicate that lack of *AtPRX71* partially suppresses the cell adhesion defect in *qua2* leaf epidermis (Figure 5). Moreover, the *esmd1* mutation completely suppressed in *qua2* both loss of epidermal cell adhesion (Verger et al., 2016) and cuticle permeability (Supplementary Figure 6), supporting the hypothesis of a link between a defective cell adhesion and an altered cuticle functionality in plants with reduced HG content. Cell separation in the hypocotyl and cotyledons of *qua1*, another mutant defective in HG (Bouton et al., 2002), can be restored by reducing water potential, indicating that adhesion defects relate to the tensile status of the tissue (Verger et al., 2018). It is conceivable that a similar correlation also occurs in *qua2*, and the observation that the *atprx71* mutation partially complements the cell adhesion defect in leaves of this mutant (Figure 5) points to a role of CW peroxidases in the ability to withstand tensile stress in the presence of a defective pectin, as *AtPRX71* also appears to negatively regulate cell expansion (Raggi et al., 2015). However, since peroxidase overexpression increases cuticle permeability also in the absence of HG defects (Survila et al., 2016; Figure 4), and plants overexpressing *AtPRX71* do not display loss of cell adhesion (Figure 5). cuticle functionality might be impaired by very high levels apoplastic peroxidases independently of its impact on cell adhesion. Moreover, as the *atprx71* mutation does not fully complement the permeability of *qua2*, peroxidase-independent mechanisms responsible for the impaired cuticle functionality of plants with defective HG can also be envisioned. It was recently proposed that, in tomato fruits, close association occurs between cutin and pectins, and the latter probably play a determinant role in the organization and formation of the cutin-polysaccharide continuum (Philippe et al., 2020), suggesting that alterations in HG content might directly impair proper assembly of cutin.

### Loss of Cuticle Functionality Is a Major Determinant of the Robust Resistance to *B. Cinerea* Caused by Altered HG Integrity

The results presented in this work indicate that the dramatic increase in cuticle permeability of plants with altered HG integrity, as well as of *fer-4* plants, is a major determinant of their robust resistance to *B. cinerea*. These conclusion is supported by the observations that both cuticle permeability and resistance in *qua2* and PG plants are suppressed by ABA treatments (Figures 1, 2) and are partially complemented in the *qua2 atprx71* double mutant (Figures 4, 6). Moreover, suppression of the cell adhesion defect in *qua2* by the *esmd1* mutation fully restores both cuticle functionality and susceptibility to *B. cinerea* (Supplementary Figure 6). On the other hand, mutations that impair different structural polysaccharides of the primary CW beside HG have no or minor effects on cuticle permeability and fungal resistance (Figures 7, 8). Our results are consistent with previous reports indicating that defects in cuticle integrity caused by overexpression of a fungal cutinase (CUTE plants) or by mutations in *Arabidopsis* genes involved in cuticle biogenesis, including *LACS2*, *LCR, FDH*, and *BDG*, confer an almost complete resistance to *B. cinerea* in rosette leaves (Bessire et al., 2007; Chassot et al., 2007; Tang et al., 2007; Voisin et al., 2009). A similar observation has also been made in cotyledons of *fad2* and *fad* triple mutant (Dubey et al., 2020).

The mechanisms by which an altered cuticle leads to increased resistance to *B. cinerea* are not fully elucidated. Different explanations have been proposed, including deregulation of cell death (Cui et al., 2019), enhanced diffusion of antifungal compounds (Bessire et al., 2007; Chassot et al., 2007), increased production and/or diffusion of DAMPs and MAMPs (Chassot et al., 2007; Curvers et al., 2010), hyperaccumulation of ROS (Asselbergh et al., 2007; L’Haridon et al., 2011), an altered leaf microbiome (Ritpitakphong et al., 2016), and over-accumulation of fungitoxic secondary metabolites on the plant surface (Dubey et al., 2020). These explanations are not mutually exclusive, and it is conceivable that multiple mechanisms contribute to the loss of susceptibility to *B. cinerea* in plants with a permeable cuticle. The results presented in this work indicate that diffusates from the *qua2* leaf surface inhibit *B. cinerea* germination and impair the ability of the fungus to successfully infect WT plants (Supplementary Figure 1), supporting the hypothesis that an enhanced accumulation and/or diffusion of antimicrobial compounds in *qua2* plants contributes to their robust resistance. Moreover, increased production and/or diffusion of elicitors are also possible, as expression of the elicitor-induced gene *PAD3* during infection occurs much earlier in *qua2* plants than in the WT (Supplementary Figure 7). It was previously shown that the ABA-hypersensitive *enhanced response to aba1-2* (*era1-2*) mutant also has a permeable cuticle and is resistant to *B. cinerea* (Cui et al., 2019). Notably, resistance in this mutant was suppressed by the *botrytis susceptible1* mutation that confers deregulated cell death (Cui et al., 2019). Since *qua2* plants show reduced Evans blue staining at late stages of fungal infection (Supplementary Figure 4), we cannot rule out that regulation of cell death might play a role in the resistance of this mutant, though it is also possible that the observed reduction in staining simply reflects the reduced extent of lesion development in the mutant.

Our results suggest that peroxidase-mediated ROS accumulation plays a major role in resistance of plants with altered HG content. Loss of *PRX71* in *qua2* reduces peroxidase activity and ROS levels (Raggi et al., 2015) and suppresses cuticle permeability and resistance (Figures 4, 6). This suggests that the resistant phenotype of plants with altered HG is due to their defective cuticle, which in turn is dependent on their enhanced peroxidase levels that increase the extent of loss of cell adhesion in the epidermis. Accumulation of high ROS levels was also observed in *Arabidopsis* plants with an altered cuticle caused by cutinase treatment or mutations in *LACS* and *BDG* and in the tomato *sitiens* mutant and linked to their resistance to *B. cinerea* (Asselbergh et al., 2007; L’Haridon et al., 2011). In cotyledons of *Arabidopsis*, enhanced ROS production could not be correlated to resistance to *Botrytis* and cuticle permeability (Dubey et al., 2020). It is however conceivable that in *Arabidopsis* and tomato leaves, a positive feedback mechanism exists between HG integrity, cuticle functionality, and peroxidase-mediated ROS production: loss of HG integrity results in elevated peroxidase levels and ROS accumulation, causing a defective cuticle that triggers further production of ROS. However, additional, cuticle-independent mechanisms by which elevated peroxidase levels may contribute to the enhanced resistance of plants with defective HG cannot be ruled out. Indeed, class III peroxidases play multiple roles in plant-pathogen interactions, mediating reinforcement of the CW, and contributing to the oxidative burst induced by DAMPs and MAMPs (Almagro et al., 2009). Antisense expression of the French bean peroxidase type 1 in *Arabidopsis* reduces the expression of two endogenous peroxidases (*PRX33* and *PRX34*) required for elicitor-induced oxidative burst and gene expression and for basal resistance to *B. cinerea* (Bindschedler et al., 2006; Daudi et al., 2012). Accumulation of ROS also mediates immunity to *B. cinerea* induced by wounding, and both wound-induced ROS accumulation and resistance are suppressed by ABA (L’Haridon et al., 2011). We indeed found that exogenous ABA in *qua2* plants reduces ROS levels (Supplementary Figure 3) and restores fungal susceptibility (Figure 2). Therefore, ROS generated by an increased expression of apoplastic peroxidases might lead, in plants with altered HG, to a robust resistance to *B. cinerea* not only because they affect cuticle integrity, but also because they might restrict fungal infection either directly or through the activation of other defense-related pathways. Notably, ROS are produced in the host apoplast by *B. cinerea* as a virulence factor, possibly mediating induction of cell death (Govrin and Levine, 2000). However, high levels of ROS *per se* do not induce cell death in *qua2* leaves (Supplementary Figure 4) and might indeed promote immunity to *B. cinerea* (Asselbergh et al., 2007; Serrano et al., 2014; Survila et al., 2016).

Previous work indicates that SA-, JA-, and ethylene-mediated responses all contribute to basal resistance of *Arabidopsis* to *B. cinerea* (Ferrari et al., 2003). Both basal- and fungal-induced expression of *PDF1.2* and *PR-1*, typical marker genes for these defense-related pathways, are comparable in WT and *qua2* plants. Similarly, microarray analysis previously failed to reveal constitutive expression of *PR-1* or *PDF1.2* in PG plants (Raggi et al., 2015). Therefore, the resistant phenotype of plants with impaired HG integrity does not appear to be dependent of the SA-, JA-, or ethylene-mediated pathways, as also previously observed for rosettes of CUTE plants (Chassot et al., 2007) and cotyledons of the *fad2* and *fad* triple mutant (Dubey et al., 2020). PAD3-mediated biosynthesis of camalexin is crucial for both basal- and elicitor-induced resistance of *Arabidopsis* to *B. cinerea* (Ferrari et al., 2003, 2007), and expression of *PAD3* in response to this pathogen and to OGs occurs independently of SA-, ethylene-, and JA-mediated signaling (Ferrari et al., 2007). Notably, basal- and pathogen-induced expression of *PAD3* is greatly enhanced in *qua2* (Supplementary Figure 7). Indeed, it was previously suggested that altered cuticle permeability in plants that overexpress class III peroxidases primes the expression of OG-responsive defense-related genes (Survila et al., 2016). However, in contrast to resistance, the enhanced expression of *PAD3* in *qua2* during early stages of infection is not suppressed by mutations in *AtPRX71* (Supplementary Figure 7). Consistently, resistance of CUTE plants to *B. cinerea* is also independent of *PAD3* (Chassot et al., 2007). Therefore, increased production of pectin-derived signals, like OGs, might promote, in plants with altered HG, earlier expression of defense responses during infection, but this has a minor impact on resistance against *B. cinerea*, compared to the effects caused by a defective cuticle.

### Cuticle Defects and Robust Resistance to *B. Cinerea* Are Specifically Associated to Loss of HG Integrity or of the Ability to Perceive it

It is now widely accepted that defects in CWI caused by a biotic or abiotic stress trigger responses aimed at restoring or compensating for the damage to ensure proper CW functionality (Gigli-Bisceglia et al., 2020; Rui and Dinneny, 2020). Increasing evidence indicates that loss of CWI also induces defenses effective against pathogens and that there is extensive crosstalk between the CWI maintenance system and PTI (Bacete et al., 2018; Bacete and Hamann, 2020; Gigli-Bisceglia et al., 2020). Indeed, mutants impaired in different CW structural components often display enhanced resistance to specific pathogens (Bacete et al., 2018; Molina et al., 2020), and increased resistance to necrotrophic fungi is often observed in mutants impaired in secondary CW components (Ellis and Turner, 2001; Hernández-Blanco et al., 2007; Molina et al., 2020). We found that neither cuticle permeability nor resistance to *B. cinerea* are significantly affected in a set of mutants impaired in different polysaccharidic components of the primary CW (Figures 7, 8), suggesting that loss of HG integrity can specifically confer a robust resistance to this pathogen, probably because of its impact on cuticle integrity. Notably, *fer-4* also shows increased cuticle permeability and strong resistance to *B. cinerea* (Figures 7, 8). This might be the consequence of a defect in HG content, like that observed in *qua2*, though a detailed analysis of pectin composition in *fer-4* leaves is required to support this conclusion. FER-dependent signaling is necessary to maintain pectin integrity during salt stress, which causes softening of the CW in roots, and cells in *fer-4* roots explode during growth recovery (Feng et al., 2018). Notably, similar defects are observed in the *mur1* mutant, which disrupts pectin cross-linking, and *fer-4* CWI defects can be rescued by calcium and borate, which facilitate HG and RGII cross-linking (Feng et al., 2018). Moreover, a significant reduction in de-esterified pectin was recently detected at the filiform apparatus of *fer-4* ovules (Duan et al., 2020). This indicates that FER might be required for sensing pectin integrity, and lack of it results in the inability of plants to adjust pectin composition in response to stress. It must be noted, however, that FER can also function as a scaffold for PAMP receptors, positively modulating immunity, and can also interact with endogenous RAPID ALKALINIZATION FACTOR propeptides to inhibit PTI (Stegmann et al., 2017). Induction of ROS and activation of MAP kinases in response to flg22 are also enhanced in *fer-4* (Keinath et al., 2010), supporting the notion that lack of FER induces the constitutive activation of signaling pathways normally activated during PTI. Further investigation will help determine if the increased resistance to *B. cinerea* of *fer-4* plants is caused by a defect in maintaining a proper pectin composition, and the consequent alteration of cuticle integrity or rather depends on the negative role of FER in modulating PTI.

## Conclusion

We have shown that loss of HG integrity, or of the ability to monitor it, results in an increase cuticle permeability that correlates with a defective epidermal cell adhesion and leads to accumulation of antimicrobial compounds on the leaf surface. Increased permeability confers an almost complete resistance to *B. cinerea*, and both phenotypes are partially dependent on *AtPRX71* and completely suppressed by loss of *ESMERALDA1*. In addition, HG alterations also appear to enhance defense responses during fungal infection independently of cuticle permeability, but these responses do not seem to play a major role in the robust resistance to *B. cinerea* observed in *qua2*. These results indicate that HG has a crucial role in maintaining cuticle integrity and highlight the complexity of the interactions between CW composition and resistance to pathogens.

## Data Availability Statement

The raw data supporting the conclusions of this article will be made available by the authors, without undue reservation.

## Author Contributions

RL, FF, GDL, CN, HJM, and SF designed the experiments and analyzed the data. RL, FF, HJM, and KG performed the experiments. RL, CN, GDL, and SF wrote the manuscript. All authors contributed to the article and approved the submitted version.

## Conflict of Interest

The authors declare that the research was conducted in the absence of any commercial or financial relationships that could be construed as a potential conflict of interest.

## Publisher’s Note

All claims expressed in this article are solely those of the authors and do not necessarily represent those of their affiliated organizations, or those of the publisher, the editors and the reviewers. Any product that may be evaluated in this article, or claim that may be made by its manufacturer, is not guaranteed or endorsed by the publisher.

## Footnotes

^1^ http://rsb.info.nih.gov/ij/

^2^ http://rsbweb.nih.gov/ij/index.html
