# Supplementary material for: Impaired Cuticle Functionality and Robust Resistance to Botrytis cinerea in Arabidopsis thaliana Plants With Altered Homogalacturonan Integrity Are Dependent on the Class III Peroxidase AtPRX71
Source: Front Plant Sci. 2021 Aug 16;12:696955. doi: 10.3389/fpls.2021.696955 (PMC8415794; doi:10.3389/fpls.2021.696955)
Supplement: Supplementary file 1 [file Data_Sheet_1.PDF]

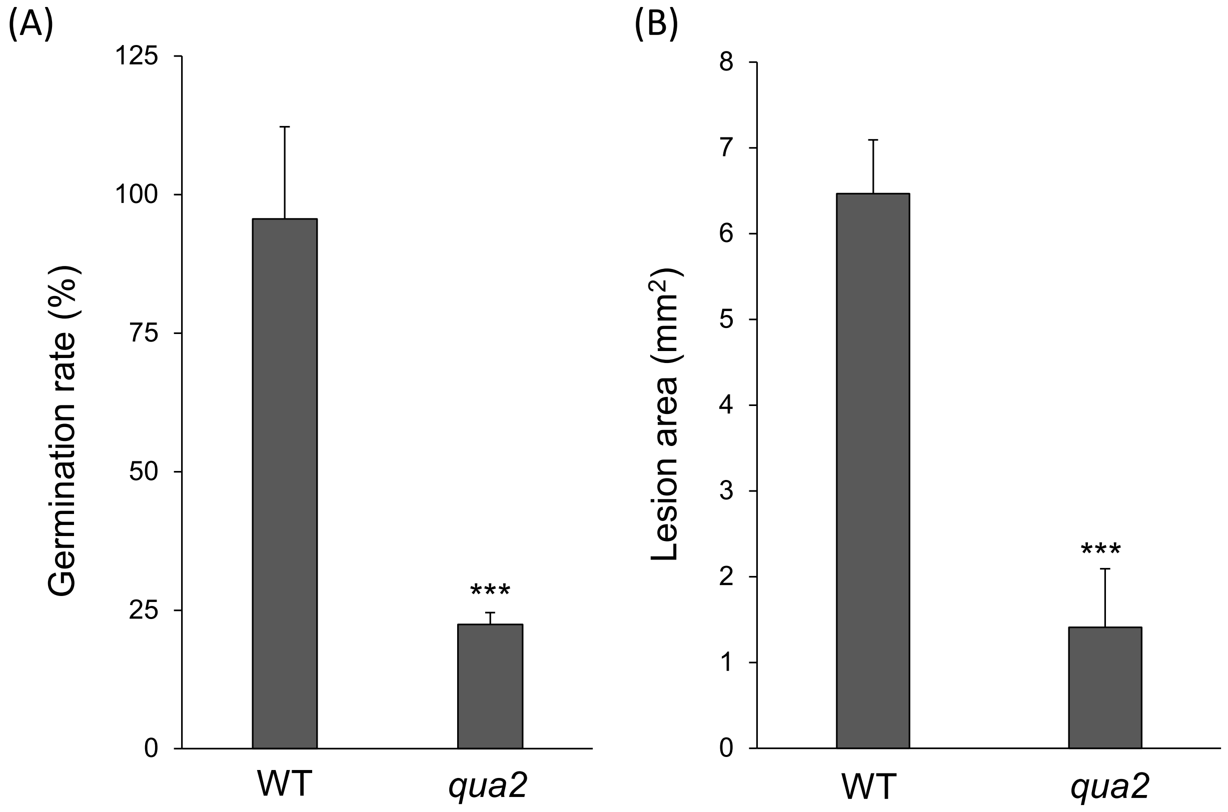

Supplementary Figure 1. Diffusates of *qua2* leaves suppress *B. cinerea* germination and infection. Potato dextrose broth droplets (5 µl) were placed on the adaxial face of rosette leaves of five-week-old plants WT or *qua2* plants in 100% humidity and, after 44 hours, the diffusates were recovered and inoculated with *B. cinerea* spores to a final concentration of 5×10^5 ml^{-1}. A, *In vitro* germination rate of *B. cinerea* spores incubated in diffusates of WT and *qua2* leaves. Bars indicate average germination rate ± SD (n=3). Asterisks indicate statistically significant differences according to Student's t-test (***, $P<0.01$). B, Rosette leaves of five-week-old WT plants were inoculated with *B. cinerea* spores suspended in diffusates from WT or *qua2* leaves. Lesion area was measured two days after inoculation (n> 10 for each genotype). Bars indicate average lesion area ± SD (n=3). Asterisks indicate statistically significant differences according to Student's t-test (***, $P<0.01$).

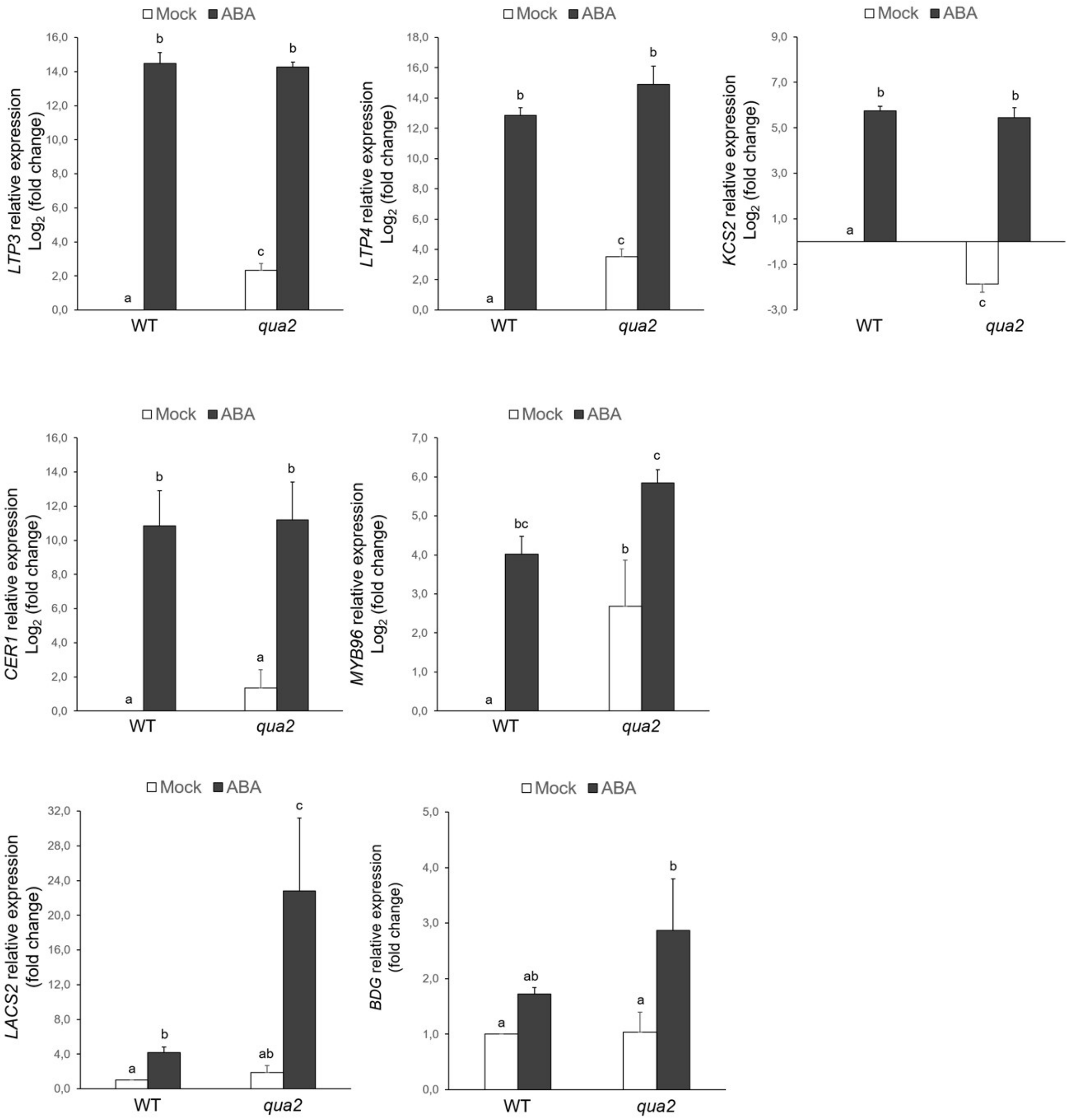

Supplementary Figure 2. **Expression of cuticle-related genes in *qua2*.** A, Expression of cuticle-related genes in fully expanded rosette leaves of five-week-old WT and *qua2* plants was evaluated after 24 h of treatment with 0.01% MeOH (mock) or 100 µM ABA. Transcript levels were quantified by qPCR using *UBQ5* as control. Data represent the mean of three biological replicates ± SE, normalized against *UBQ5* and relative to control-treated WT. Different letters indicate statistically significant differences, according to two-way ANOVA followed by Tukey's significance test (P<0.05).

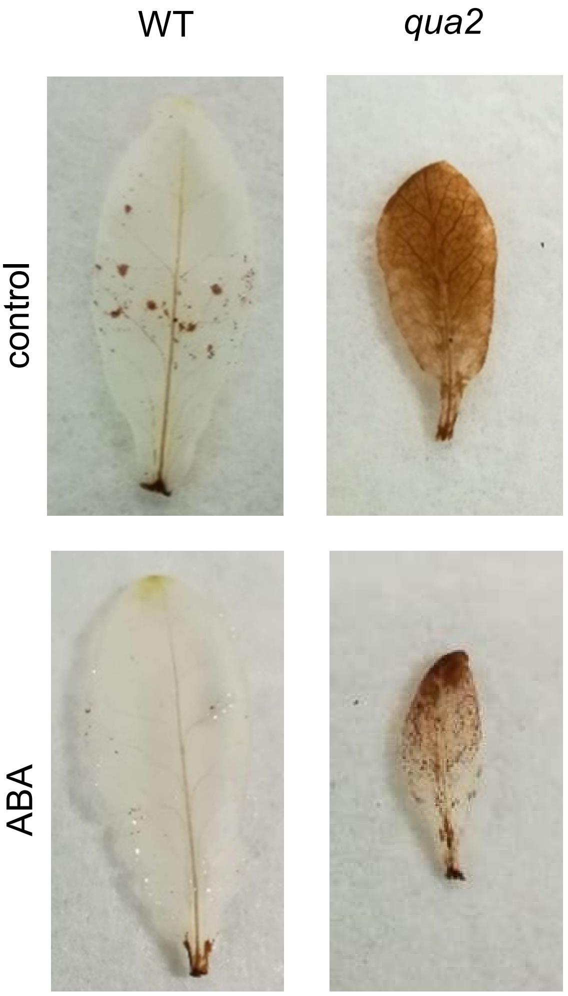

Supplementary Figure 3. Exogenous ABA partially suppresses ROS accumulation in *qua2*. WT and *qua2* five-week-old plants were sprayed with 0.01% MeOH (control) or 100 µM ABA and, after 24 h, leaves were harvested, and ROS were stained with DAB.

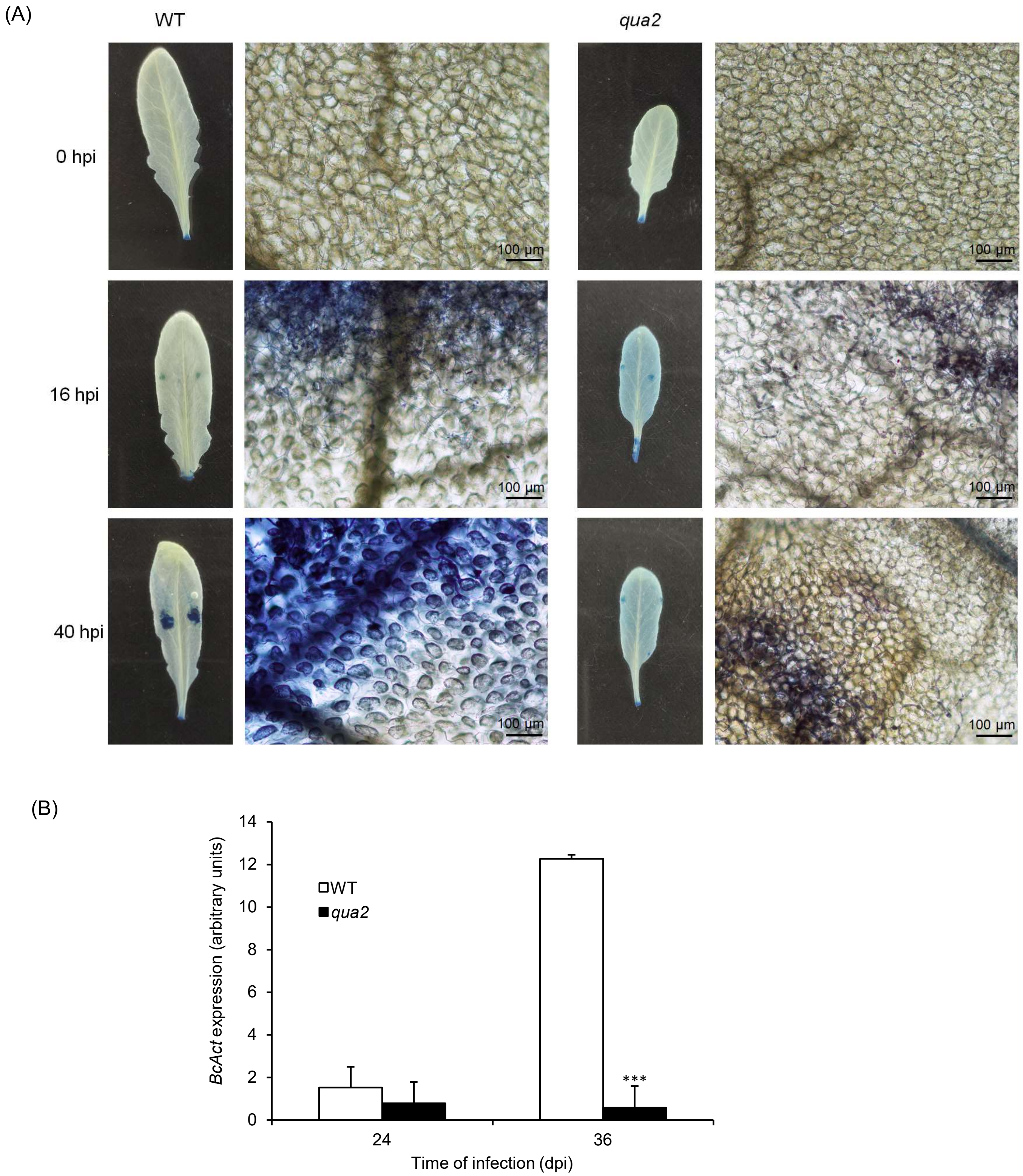

Supplementary Figure 4. Reduced cell death and fungal gene expression in *qua2* leaves infected with *B. cinerea*. A) WT and *qua2* Arabidopsis five-week-old leaves were stained for cell death with Evans blue. Images are representative of at least two leaves from three independent plants. Right panels show details of the same leaves at greater magnification, in correspondance of the margin of the inoculated site. B) qPCR analysis of the expression of *BcAct*, relative to the Arabidopsis *UBQ5*, in WT and *qua2* rosette leaves inoculated with *B. cinerea* at the indicated times. Bars are mean expression ± SD (n=3). Asterisks indicate statistically significant differences between WT and *qua2*, according to Student's t-test (***, P<0.001).

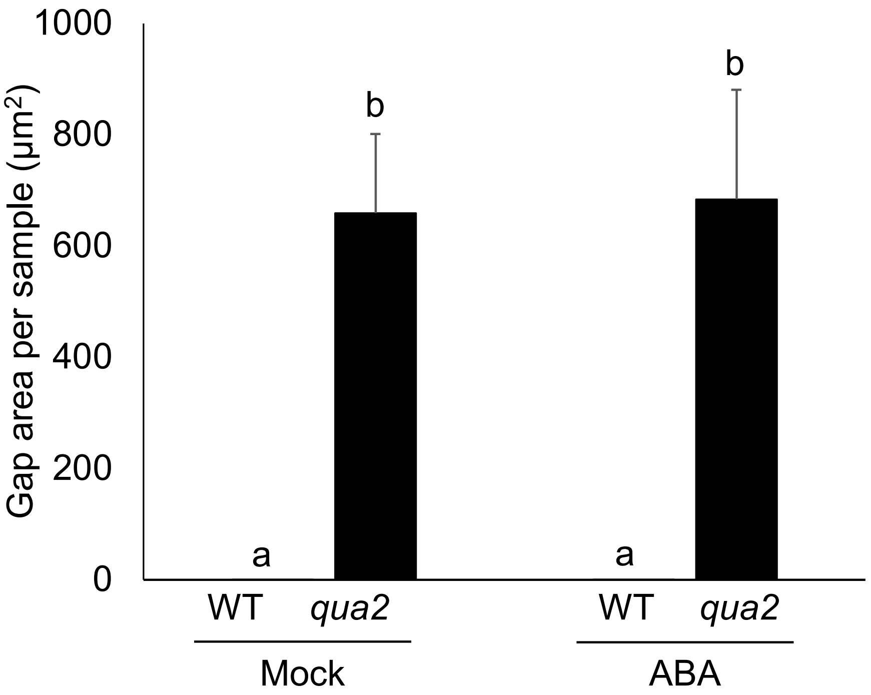

Supplementary Figure 5. Cell adhesion defects in *qua2* leaves are not suppressed by ABA. Rosette leaves of five-week-old Col-0 (WT, white bars) and *qua2* (black bars) were treated with 0.01% MeOH or 100 μM ABA and, after 24 h, plants were stained with ruthenium red and total area of the gaps between adjacent cells per image was analyzed (total area of images = 60000 μm2). Bars indicate average gap area ± SD (n=3). Letters indicate statistically significant differences, according to two-way ANOVA followed by Tukey's significance test ($P<0.05$). These experiments were repeated three times with similar results.

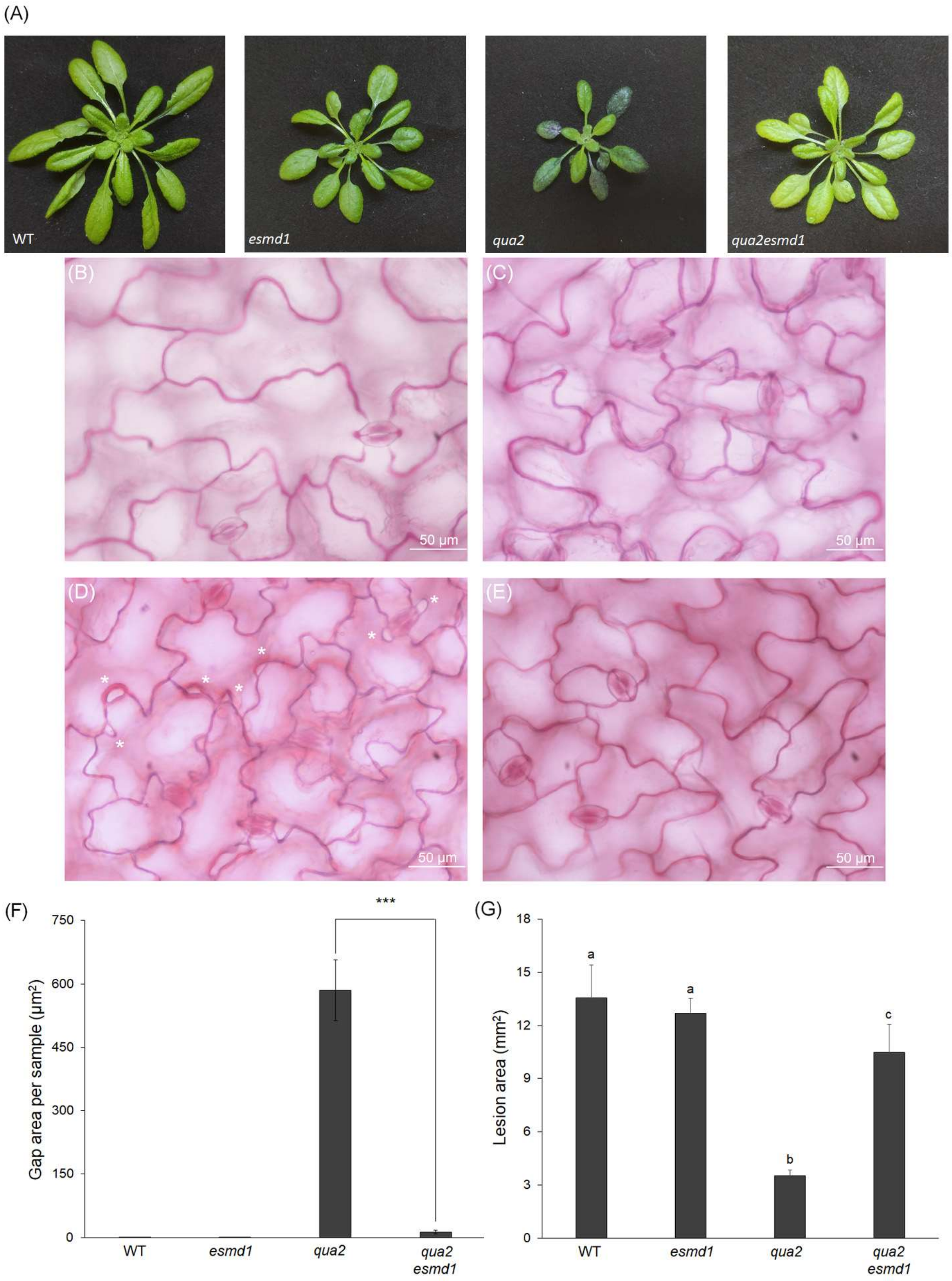

Supplementary Figure 6. Leaf cuticle permeability and resistance to *B. cinerea* in *qua2* are suppressed by the *esmd1* mutation. A, five-week-old Col-0 (WT), *qua2*, *esmd1 and qua2 esmd1 seedlings* were stained with toluidine blue for cuticle permeability. Images show a representative rosette for each genotype. WT (B), *esmd1* (C), *qua2* (D) and *qua2 esmd1* (E) leaves were stained with ruthenium red and epidermal cells of the adaxial face were photographed. Asterisks indicate gaps between adjacent cells. Representative images of at least five images per genotype. F, For each experiment, total area of the gaps between adjacent cells per image was analyzed (total area of images = 60000 μm^2). Bars indicate average gap area ± SD (n=3). Asterisks indicate statistically significant difference between *qua2* and *qua2 esmd1*, according to Student's t-test (***, $P<0.01$). (G) Rosette leaves of five-week-old Col-0 (WT), *qua2, esmd1* and *qua2 esmd1* plants were inoculated with a *B. cinerea* spore suspension, and lesion area was measured two days after inoculation (n> 10 for each genotype). Bars indicate average lesion area ± SE. Different letters indicate statistically significant differences, according to one-way ANOVA followed by Tukey's significance test ($P<0.01$).

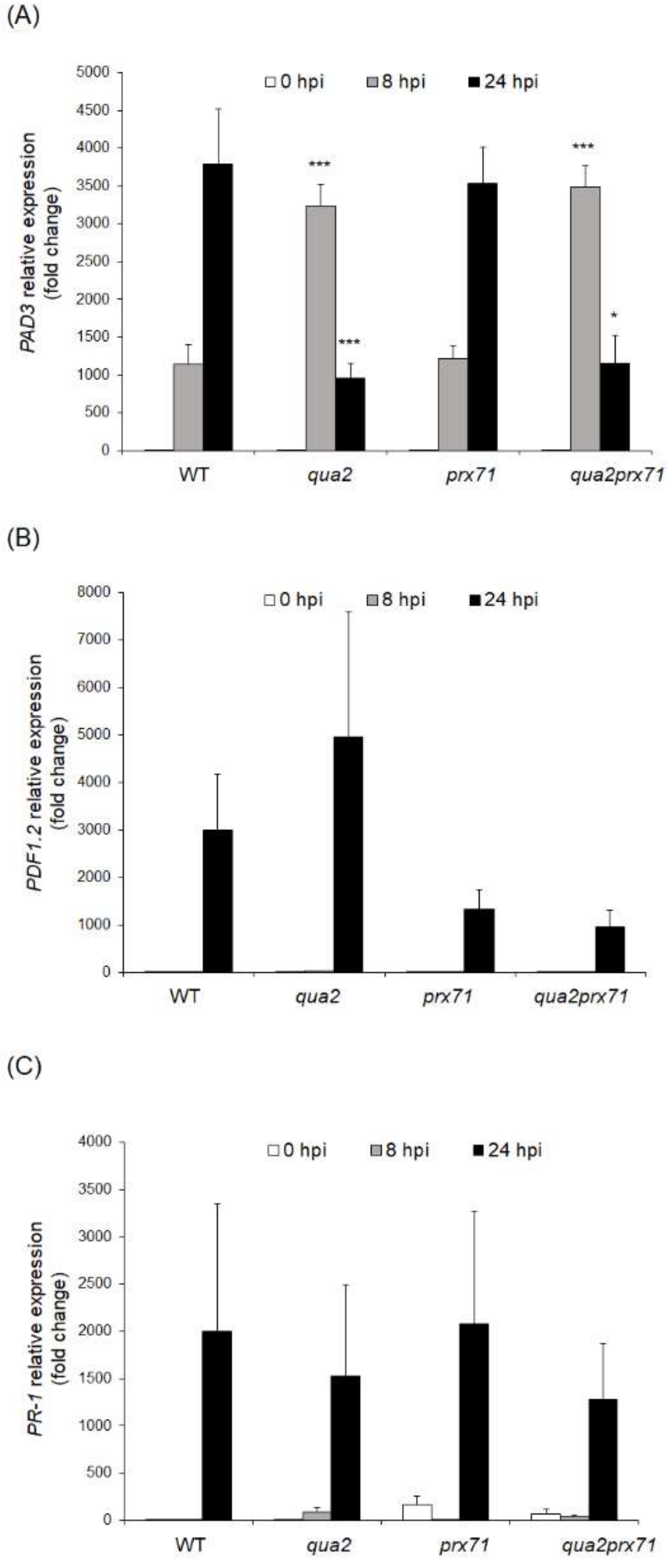

Supplementary Figure 7. Expression of defense genes in *qua2* plants during B. *cinerea* infection. *PAD3* (A) *PR-1* (B) and *PDF1.2* (C) transcript levels in rosette leaves of five-week-old WT, *qua2, atprx71* (*prx71*) and *qua2 atprx71* (*qua2prx71*) plants were quantified by qPCR using *UBQ5* as control. Data represent the mean of three biological replicates ± SD, normalized against *UBQ5* and relative to WT at 0 hpi. Asterisks indicate significant differences, according to Student's t-test, between WT and mutant plants at each time point (*, P<0.05; ***, P<0.01). No significant differences between WT and mutants were observed in (B) and (C) at any timepoint.

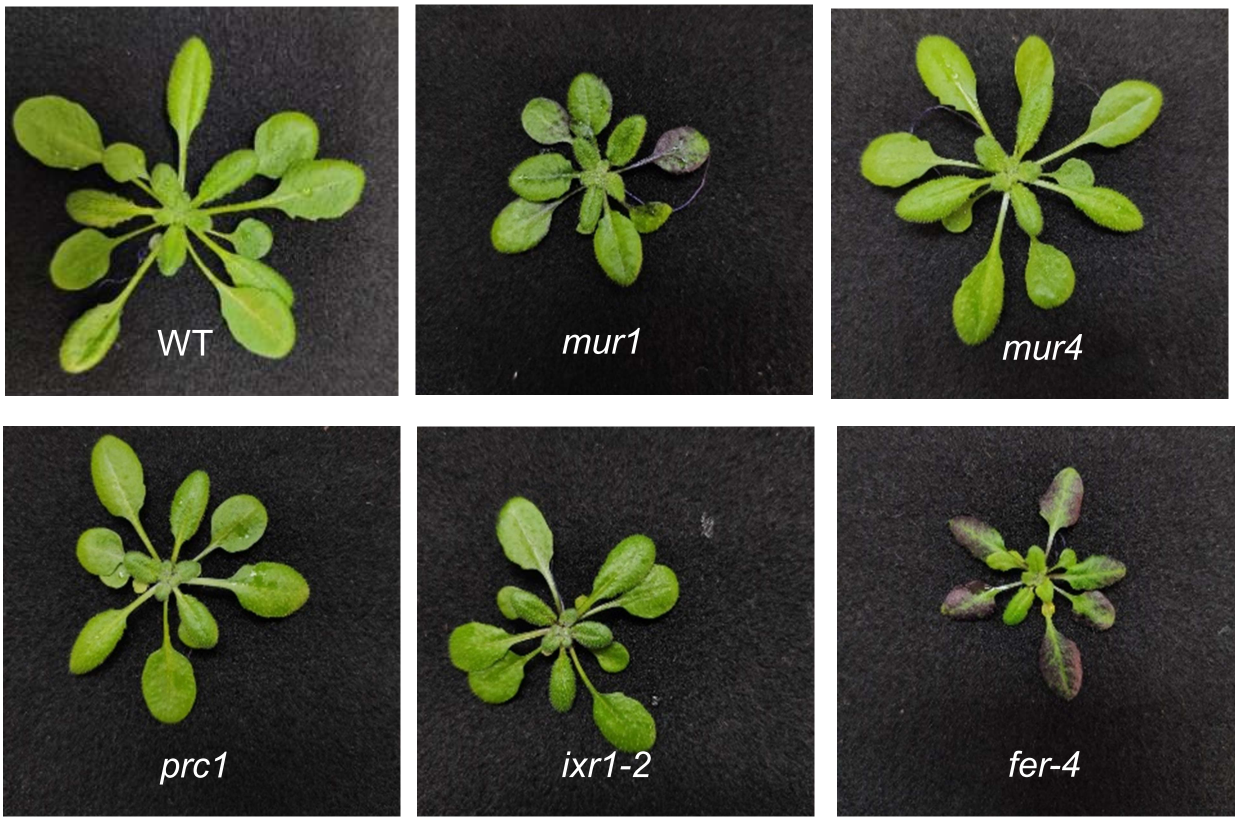

Supplementary Figure 8. Leaf cuticle permeability in whole rosettes of cell wall mutants. Rosettes of five-week-old Col-0 (WT), *mur1*, *mur4*, *prc1*, *irx1-2* and *fer-4* plants were stained with toluidine blue for cuticle permeability. Representative images of at least five plants are shown.
